# Gut *Enterococcus faecium* derived leucic acid mediates the antiobesity effect of dietary fiber

**DOI:** 10.1002/imt2.70173

**Published:** 2026-09-21

**Authors:** Baifei Hu, Qianru Gao, Junmin Hui, Mengqiong Wang, Haiyan Gao, Kai Li, Junping Zheng, Zhe Dai, Songlin Liu, Hongtao Liu

**Affiliations:** ^1^ College of Basic Medical Sciences; Key Laboratory of Chinese Medicinal Resource and Chinese Herbal Compound of the Ministry of Education Hubei University of Chinese Medicine Wuhan China; ^2^ Hubei Shizhen Laboratory Wuhan China; ^3^ Department of Endocrinology Zhongnan Hospital of Wuhan University Wuhan China

**Keywords:** dietary fiber, *Enterococcus faecium*, leucic acid, obesity, oxidative phosphorylation

## Abstract

Obesity is a globally prevalent metabolic disorder closely associated with gut microbiota dysbiosis. However, the precise roles of gut microbiota and microbial metabolism in this disease remain unclear. Through fecal metagenomic data mining and clinical cohort validation, we identified a gut bacterial strain with anti‐obesity potential, *Enterococcus faecium* (*E. faecium*). It inhibited adipocyte enlargement, dyslipidemia, and hepatic steatosis in obese mice by metabolizing leucine to produce leucic acid (LEA). Colonization with *E. faecium* or exogenous LEA supplementation improved obesity‐associated metabolic phenotypes. Mechanistically, *E. faecium* metabolized leucine into LEA via putative branched‐chain amino acids metabolic enzymes. The bacterium‐derived LEA regulated mitochondrial oxidative phosphorylation and thus alleviated obesity. Furthermore, using a high‐content screening system and an in vitro fermentation model, we found that the dietary fiber glucomannan exerted anti‐obesity effects by promoting the growth of *E. faecium*. This study reveals the critical role of microbial metabolism and LEA production in ameliorating the onset and progression of obesity, providing a theoretical basis for dietary fiber intervention strategies targeting the gut microbiota.

## Highlights

- Metagenomic and clinical data verified *E. faecium* as an anti‐obesity gut bacterium.
- *E. faecium* alleviated obesity via leucine conversion to leucic acid (LEA).
- LEA modulated mitochondrial oxidative phosphorylation against obesity.
- Glucomannan inhibited obesity by enriching *E. faecium* for microbiota intervention.

## Graphical abstract

This study identified *Enterococcus faecium* as a gut bacterium with anti‐obesity activity, which alleviated adipocyte hypertrophy, dyslipidemia, and hepatic steatosis by converting leucine into leucic acid (LEA) to regulate mitochondrial oxidative phosphorylation. In addition, dietary glucomannan effectively enriched this bacterium, providing a theoretical basis for developing precision dietary fiber‐based anti‐obesity strategies targeting the gut microbiota.

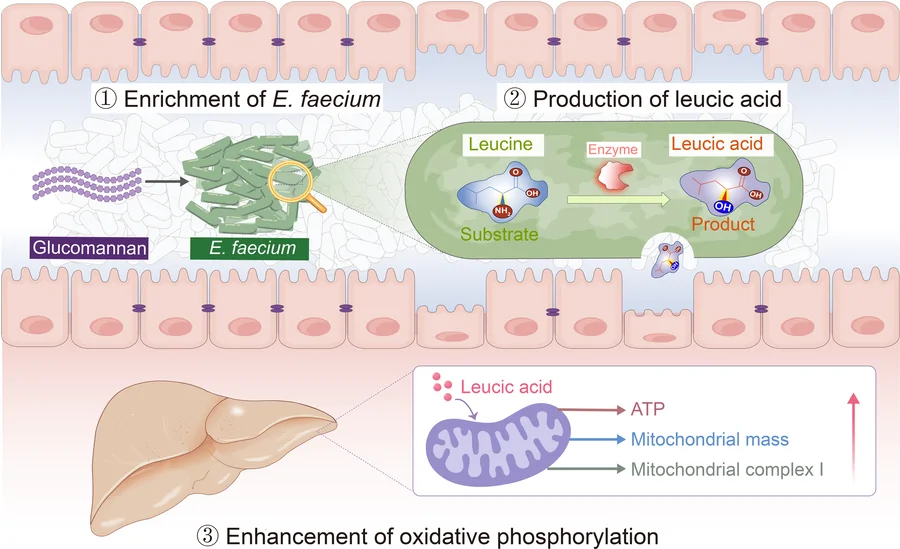

## INTRODUCTION

The World Health Organization (WHO) defines obesity as the abnormal or excessive fat accumulation that threatens health. The global prevalence of obesity continues to increase and has become a major threat to human health in the 21st century [1, 2]. Currently, approximately 2.11 billion adults and 174 million adolescents worldwide are experiencing overweight and obesity [3, 4], and nearly half of adults in the United States are predicted to develop obesity by 2030 [5, 6]. Obesity is mainly caused by long‐term energy metabolism imbalance and abnormal ectopic fat accumulation [7, 8]. It is highly correlated with various metabolic diseases such as coronary heart disease and diabetes, resulting in enormous public health and economic burdens. Therefore, it is urgent to develop safe and effective intervention strategies [9]. The human gut microbiota consists of trillions of symbiotic microorganisms and plays a core regulatory role in the occurrence and progression of obesity and related metabolic disorders [10, 11, 12]. Gut microbiota produces a variety of physiologically active substances, including health benefits such as anti‐inflammatory, analgesic, and antidepressant products, as well as potentially harmful substances such as endotoxins, carcinogens, and immunotoxins, which can enter the circulation to promote or inhibit the development of obesity [13, 14]. For example, gut microbiota‐derived homovanillic acid could alleviate depressive phenotypes [15], and nicotinic acid produced by *Parabacteroides distasonis* effectively improves insulin resistance [16]. Microbial metabolites are the bridge between microbiota and host obesity, and microbial metabolites may be better targets for characterizing or controlling obesity [17].

Elevated levels of branched‐chain amino acids (BCAAs), including leucine, isoleucine, and valine, represent typical metabolic signatures of obesity and type 2 diabetes [18, 19]. Gut microbes can directly utilize and metabolize host‐derived BCAAs to maintain systemic metabolic homeostasis. Specifically, *Parabacteroides johnsonii* metabolizes valine into isobutyric acid to preserve intestinal barrier integrity [20], whereas *Ruminococcus gnavus* utilizes leucine to synthesize isovaleric acid and exert cardiovascular protective effects [21]. In addition to the above‐mentioned branched‐chain fatty acids, BCAAs can also be converted into branched‐chain α‐hydroxy acids and other metabolites through alternative pathways, among which leucic acid (LEA) derived from leucine is a potential signaling molecule [22]. However, current research on whether LEA affects the pathophysiology of obesity through gut microbiota‐host interactions remains limited and urgently needs in‐depth investigation. Dietary fiber is an excellent regulator of gut microbiota. WHO and the Codex Alimentarius Commission define dietary fiber as all carbohydrates that are neither digested nor absorbed in the small intestine and have a degree of polymerization of 10 or more monomeric units [23]. Soluble dietary fibers mainly include xyloglucans, arabinoxylans, and pectins [24, 25]. Fermented by anaerobic microbiota in the colon, dietary fiber generates abundant metabolites such as short‐chain fatty acids and branched‐chain fatty acids, which participate in the regulation of host energy metabolism through the gut–brain, gut–liver, and gut–adipose axes [26, 27, 28, 29]. For example, inulin regulates *Parabacteroides distasonis* to produce pentadecanoic acid, thereby ameliorating non‐alcoholic steatohepatitis [30]. Dietary fiber selectively enriches beneficial intestinal bacteria and remodels microbial composition [31]. Regulation of gut microbiota by dietary fiber is an effective intervention strategy for obesity.

In this study, analysis of two public datasets revealed that the abundance of *Enterococcus faecium* (*E. faecium*) was significantly reduced in obese patients, and this strain possesses the potential to be enriched by dietary fiber. This characteristic was further validated in our self‐established clinical cohort. Supplementation with *E. faecium* improved glucolipid metabolic disorders in obese mice by metabolizing leucine into LEA. As a key metabolite of *E. faecium*, LEA regulated mitochondrial oxidative phosphorylation, exerting effects similar to those of *E. faecium*, thereby inhibiting adipocyte enlargement, dyslipidemia, and hepatic steatosis in obese mice. Furthermore, using a high‐content screening system and an in vitro fermentation model, we demonstrated that the dietary fiber glucomannan (GM) promoted the growth of *E. faecium* and its anti‐obesity efficacy was confirmed in animal experiments. In summary, this study clarifies the role of *E. faecium* and its key metabolite LEA as protective factors against obesity, elucidates the regulatory axis of “dietary fiber‐*E. faecium*‐LEA‐mitochondrial oxidative phosphorylation,” and provides a strategy for gut microbiota‑based interventions in metabolic diseases.

## RESULTS

### *E. faecium* was the candidate strain of dietary fiber for obesity prevention

**FIGURE 1:**
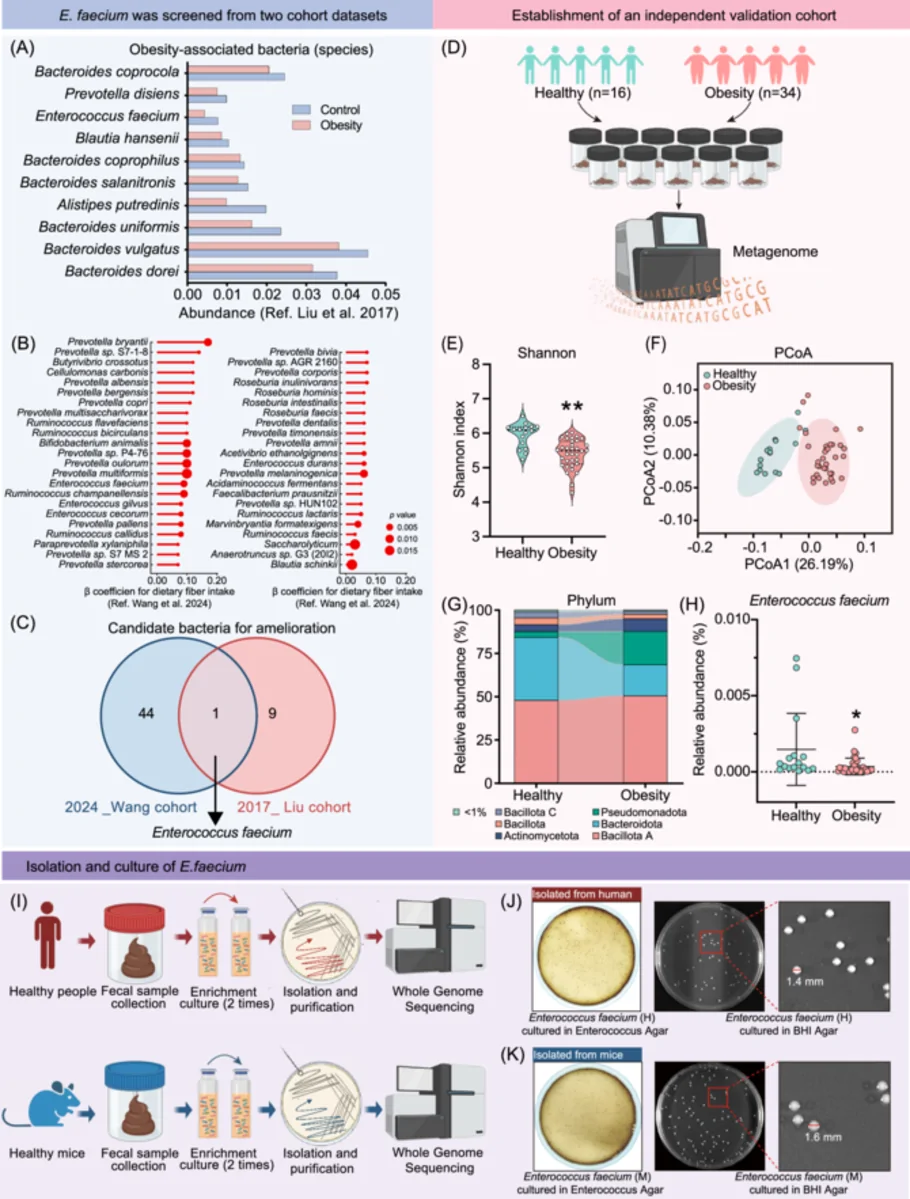
Screening of candidate strains for obesity prevention with dietary fiber. (A) Obesity‐related bacterial species are enriched in healthy individuals. (B) Bacterial species are enriched by the intake of dietary fiber. (C) Intestinal species associated with both obesity and dietary fiber. (D) Experimental scheme of metagenomic sequencing. (E) Shannon index of the healthy and obesity groups. (F) Principal co‐ordinates analysis of the healthy and obesity groups. (G) Stacked bar chart of microbial relative abundance at the phylum level. (H) The relative abundance of *Enterococcus faecium* (*E. faecium*) between healthy and obesity groups. (I) Experimental design for isolation of *E. faecium* from mice and human feces. (J, K) Analysis of the morphology of *E. faecium*. Data were expressed as mean ± SD. *p < 0.05, **p < 0.01, versus healthy group.

We retrieved published fecal metagenomic datasets of obese individuals from the 2017 Liu cohort (PRJEB12123) [32], and screened out the top 10 species enriched in healthy controls but depleted in obese subjects, including *Bacteroides coprocola*, *Prevotella disiens*, *E. faecium*, and *Blautia hansenii* (Figure 1A and Table S1). Meanwhile, a total of 45 species positively correlated with dietary fiber intake were identified from the 2024 Wang cohort (QIITA, ID 11666) [33] (Figure 1B and Table S2). Venn diagram analysis revealed that *E. faecium* was the intersection species of the two cohorts, suggesting that the decreased abundance of *E. faecium* was correlated with the progression of obesity, and its abundance could be enriched by dietary fiber supplementation (Figure 1C). To verify this correlation, we established an independent validation cohort consisting of 16 healthy subjects and 34 obese patients, and performed metagenomic sequencing on fecal samples (Figure 1D). In terms of alpha diversity, the Shannon, Chao1, and ACE indices of gut microbiota in obese patients were significantly lower than those in healthy individuals, indicating a remarkable decline in gut microbial diversity under obese conditions (Figure 1E and Figure S1A). Principal coordinate analysis (PCoA), non‐metric multidimensional scaling (NMDS), and orthogonal partial least squares discriminant analysis (OPLS‐DA) all demonstrated significant differences in gut microbiota composition between the Healthy and Obesity groups. PCoA1 and PCoA2 explained 26.19% and 10.38% of the intergroup variation, respectively (Figure 1F and Figure S1B,C). As shown in Figure 1G, the abundance of Bacteroidota was markedly decreased while that of Firmicutes was significantly increased in the Obesity group, leading to an elevated Firmicutes/Bacteroidota ratio compared with the Healthy group, which was consistent with the typical gut microbial alteration pattern in obese populations. The heatmap of differential species clearly exhibited the discrepancy in bacterial abundance between the two groups (Figure S1D). A volcano plot combined with variable importance in the projection (VIP) analysis identified 84 upregulated and 784 downregulated species in the Obesity group (Figure S1E,F). Among them, *E. faecium* was a markedly depleted key species with a high VIP value. Further abundance validation confirmed that the relative abundance of *E. faecium* was significantly reduced in obese patients (Figure 1H). Based on the above results, we isolated human‐derived *E. faecium* (H) and mouse‐derived *E. faecium* (M) from fecal samples of healthy humans and mice, respectively (Figure 1I). Strains were identified via enrichment culture, isolation and purification, as well as whole‐genome sequencing, and their colony morphology are shown in Figure 1J,K. In conclusion, *E. faecium* serves as a candidate strain of dietary fiber for obesity prevention.

### *E. faecium* alleviated the pathological phenotypes of high‐fat diet (HFD)‐induced obesity

**FIGURE 2:**
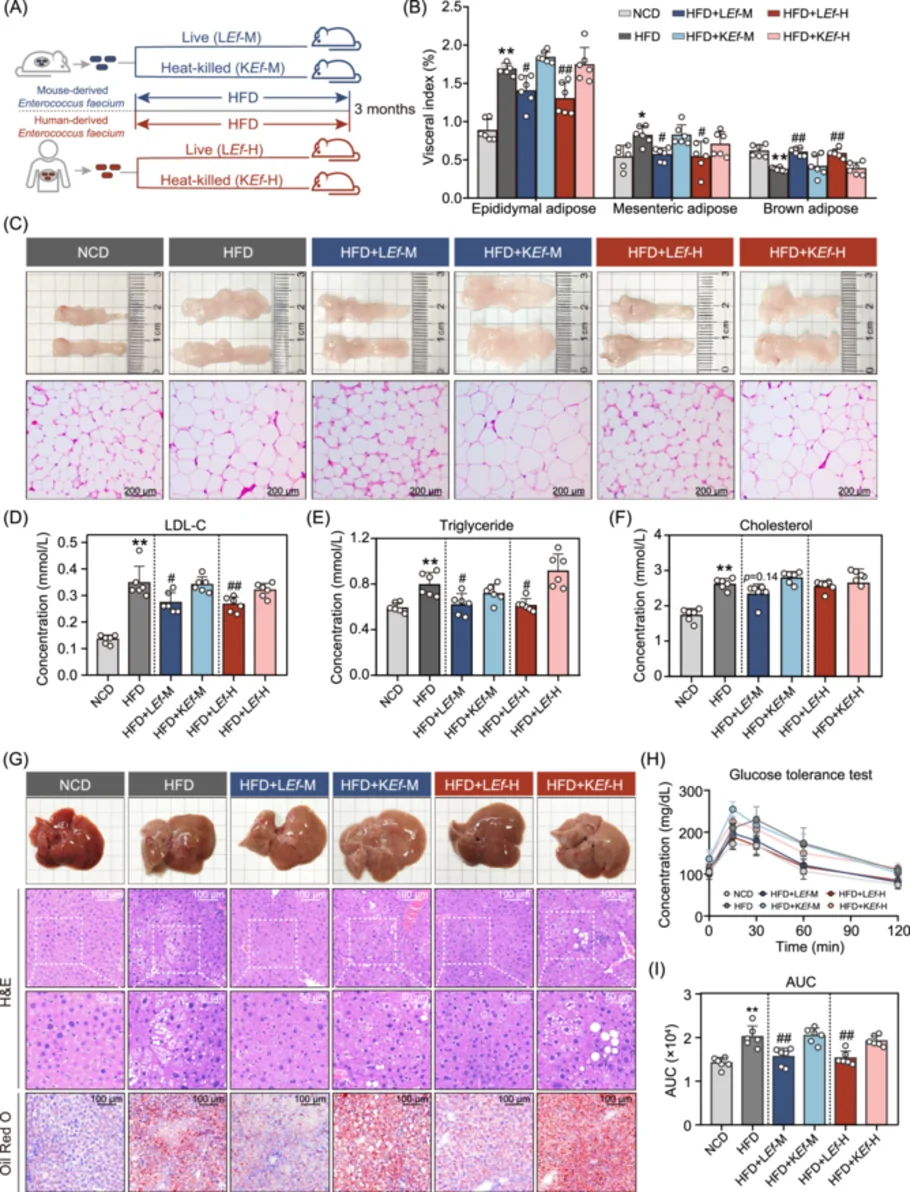
Human‐derived and mouse‐derived *E. faecium* improved HFD‐induced obesity in mice. (A) Schematic diagram. (B) Index of epididymal adipose, mesenteric adipose, and brown adipose. (C) Hematoxylin and eosin (H&E)‐stained pictures of epididymal adipose. Scale bar = 200 µm. (D–F) Concentrations of low‐density lipoprotein cholesterol (LDL‐C), triglyceride (TG), and cholesterol. (G) H&E staining and Oil Red O staining images of liver tissues. (H) Glucose tolerance test (GTT) curves. (I) Area under the curve (AUC) of GTT. Data were expressed as mean ± SD (*n* = 6–8). *p < 0.05, **p < 0.01 versus NCD group; ^#^ *p* < 0.05, ^##^ *p* < 0.01 versus HFD group. HFD, high‐fat diet; K*Ef*‐H, heat‐killed human‐derived *E. faecium*; K*Ef*‐M, heat‐killed mouse‐derived *E. faecium*; L*Ef*‐H, live human‐derived *E. faecium*; L*Ef*‐M, live mouse‐derived *E. faecium*; NCD, normal chow diet.

To investigate the role of *E. faecium* in the development of obesity, we first performed whole‐genome characterization of two isolated *E. faecium* strains. The results indicated that the two *E. faecium* strains were highly similar in genome size and GC content, but exhibited variations in MLST sequence types, plasmid sizes, and the number of coding sequences. Their core genes were enriched in key pathways such as metabolism and ABC transporters, indicating that the strains have considerable metabolic potential and laying a molecular foundation for subsequent functional validation (Figure S2A–F and Table S3). As shown in Figure 2A, mice were fed an HFD for 3 months and given human‐derived and mouse‐derived live *E. faecium* or heat‐killed *E. faecium* daily to determine the role of *E. faecium* in the development of obesity. First, we determined the content of *E. faecium* in feces using qPCR, and the results showed that live *E. faecium* successfully colonized the intestine (Figure S3A–C). As shown in Figure S3D,E, the body weight of the HFD group increased by 12.8% compared with the normal chow diet (NCD) group at the end of the experimental period, indicating successful establishment of the obesity model. Supplementation with mouse‐derived live *E. faecium* (L*Ef*‐M) and human‐derived live *E. faecium* (L*Ef*‐H) significantly reduced body weight gain, visceral index of epididymal and mesenteric adipose, and increased the brown adipose (Figure 2B). Apparent morphology and Hematoxylin and eosin (H&E) staining of epididymal adipose also showed consistent results (Figure 2C). The two live *E. faecium* strains effectively improved dyslipidemia in HFD mice by reducing the concentrations of low‐density lipoprotein cholesterol (LDL‐C) and triglyceride (TG), while cholesterol levels showed a non‐significant downward trend (Figure 2D–F). Notably, H&E and Oil Red O staining showed that the degree of hepatic steatosis in obese mice was alleviated after administration of L*Ef*‐M and L*Ef*‐H (Figure 2G). Meanwhile, oral administration of two live strains of *E. faecium* improved HFD‐induced glucose intolerance in mice (Figure 2H,I). To further elucidate how *E. faecium* ameliorates hepatic lipid accumulation, we examined the expression and phosphorylation levels of key proteins in the hepatic lipid synthesis pathway. The results showed that, compared with the HFD group, *E. faecium* intervention significantly restored total AMPK protein levels and promoted its phosphorylation‐mediated activation, accompanied by increased ACC phosphorylation and downregulated SREBP1 expression (Figure S3F,G). These findings indicated that *E. faecium* suppresses hepatic de novo lipogenesis by activating the AMPK‐ACC‐SREBP1 signaling axis, providing a mechanistic basis for its anti‐obesity effect. Furthermore, we observed that both human‐derived and mouse‐derived *E. faecium* had an ameliorative effect on obesity. This ameliorative effect depended on the activity of *E. faecium* rather than the bacteria themselves.

**FIGURE 3:**
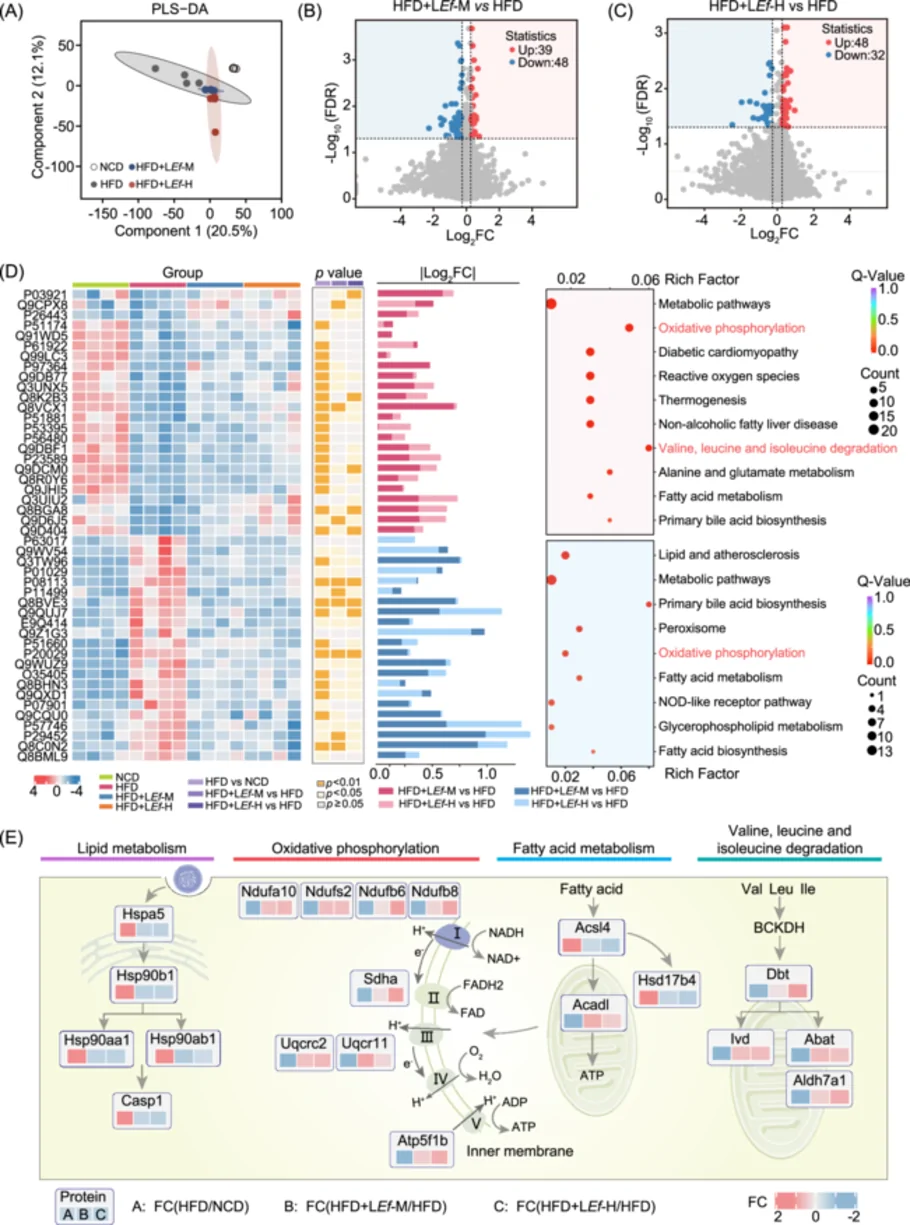
*E. faecium* regulated hepatic proteomics in HFD‐induced obese mice. (A) Partial least squares discriminant analysis (PLS‐DA) diagram. (B, C) Volcano plots of differentially expressed proteins. (D) Clustering heatmap of differentially expressed proteins. (E) Analysis of protein expression in key pathways.

Furthermore, proteomics was applied in the study to investigate the regulatory effects of *E. faecium* intervention on the hepatic protein profiles of obese mice induced by HFD. PLS‑DA analysis revealed clear separation in protein expression profiles among the four groups, indicating that *E. faecium* intervention could effectively modulate the hepatic protein profile in HFD mice (Figure 3A). With the thresholds of FC > 1.2 and FDR < 0.05 for screening differentially expressed proteins (DEPs), the volcano plots showed that 87 DEPs (39 upregulated and 48 downregulated) were identified in L*Ef*‐M group, while 80 DEPs (48 upregulated and 32 downregulated) were detected in L*Ef*‐H group (Figure 3B,C). The clustering heatmap combined with *p*‑value and |log_2_FC| distribution illustrated distinct expression patterns and statistical significance across groups (Figure 3D, left and middle). Functional enrichment analysis further demonstrated that the regulated proteins were mainly enriched in metabolic pathways, oxidative phosphorylation, and BCAA degradation (Figure 3D, right). Analysis of key pathway proteins indicated that HFD markedly upregulated the expression of lipid metabolism‐related proteins such as Hspa5 and Hsp90 family members, disrupted hepatic lipid homeostasis, and inhibited the expression of mitochondrial electron transport chain core subunits (e.g., Ndufa10 and Sdha) (Figure 3E). The two *E. faecium* strains restored the abnormal expression of the above proteins and alleviated mitochondrial dysfunction. In addition, HFD downregulated key enzymes of fatty acid β‐oxidation (e.g., Acadl) and proteins involved in BCAAs catabolism (e.g., Dbt and Ivd), hindering normal metabolism. Intervention with L*Ef*‐M and L*Ef*‐H reversed these expression disorders and recovered hepatic metabolic homeostasis. To further verify the oxidative phosphorylation pathway enriched by proteomics analysis, functional validation was performed. As presented in Figure S4A and S4B, compared with the NCD group, the activity of mitochondrial respiratory chain complex I and hepatic ATP content were markedly reduced in the HFD group. L*Ef‐*M and L*Ef*‐H interventions significantly restored complex I activity and elevated ATP levels, which confirmed that *E. faecium* effectively ameliorated mitochondrial energy metabolism abnormalities induced by HFD.

### LEA was identified as a functional candidate produced by *E. faecium*

**FIGURE 4:**
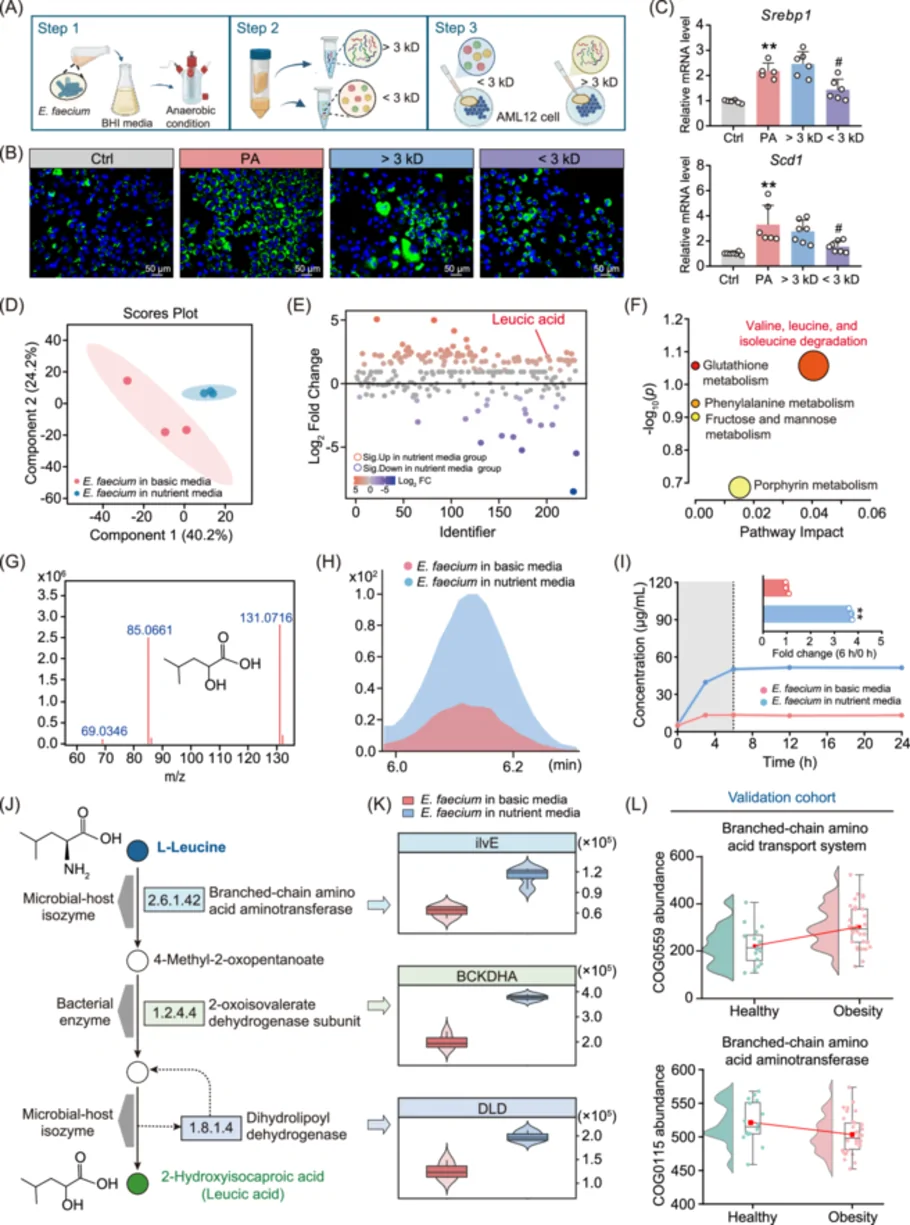
Identification of leucic acid (LEA) as a key bioactive metabolite produced by *Enterococcus faecium* (*E. faecium*). (A) Schematic diagram. (B) Lipid droplet accumulation in AML12 cells. Scale bar = 50 µm. (C) Relative mRNA expression levels of *Srebp1* and *Scd1* in AML12 cells. (D) PLS‐DA of untargeted metabolomics. (E) Volcano plot of differential metabolites. (F) KEGG pathway analysis. (G) Mass spectrometry identification of LEA. (H) Extracted ion chromatograms. (I) Time‐course analysis of LEA production. (J) Proposed biosynthetic pathway of LEA. (K) Relative abundance of three putative key enzymes involved in LEA synthesis. (L) Metagenomic analysis of BCAAs‐related genes. *p < 0.05, **p < 0.01 versus Ctrl group; ^#^ *p* < 0.05 versus PA group.

To screen the key bioactive components responsible for the lipid metabolism‐regulating effects of *E. faecium*, the culture supernatant of *E. faecium* was separated into high‐molecular‐weight (>3 kDa) and low‐molecular‐weight (<3 kDa) fractions in the study, which were then applied to a palmitic acid (PA)‐induced AML12 hepatocyte model (Figure 4A). The low‐molecular‐weight fraction exhibited superior efficacy in ameliorating PA‐induced lipid droplet accumulation and downregulating the expression of lipid metabolism‐related genes (*Srebp1* and *Scd1*), suggesting that the bioactive components were enriched in the low‐molecular‐weight fraction (Figure 4B,C).

To identify the key metabolite, untargeted metabolomics analysis was performed on *E. faecium* cultured in basal and nutrient media. The results showed a significant separation in metabolic profiles between the two culture conditions (Figure 4D and Figure S5A). Heatmap and volcano plot analyses indicated that LEA was markedly upregulated and accumulated at a higher level in the nutrient medium group (Figure S5B and Figure 4E). KEGG enrichment analysis suggested that the BCAA degradation pathway might be involved in the related metabolic regulation (Figure 4F). Mass spectrometry identification and abundance comparison verified that the LEA abundance was substantially higher in the nutrient medium group than in the basal medium group (Figure 4G–H). Time‑course detection revealed that after 6 h of culture, the production of LEA in the nutrient medium was approximately fourfold higher than that in the basal medium group, which confirmed that LEA was a characteristic metabolite abundantly synthesized by *E. faecium* (Figure 4I).

KEGG pathway annotation indicated that LEA is a metabolic derivative of leucine, synthesized by *E. faecium* through the catalysis of BCAAs transaminase (IlvE, EC 2.6.1.42), 2‐oxoisovalerate dehydrogenase subunit (BCKDHA, EC 1.2.4.4), and dihydrolipoamide dehydrogenase (DLD, EC 1.8.1.4) (Figure 4J). Consistently, proteomic analysis revealed a distinct separation in the protein expression profiles of *E. faecium* cultured in basal versus nutrient media, with the abundances of these three key enzymes significantly upregulated in the nutrient medium group (Figure 4K and Figure S5C,D). Furthermore, metagenomic analysis of clinical cohorts demonstrated that the abundance of BCAAs degradation‐related transaminase genes was markedly reduced in the gut microbiota of obese patients, whereas genes associated with the BCAAs transport system were significantly elevated (Figures 4L and S5E). These findings suggest that the BCAAs metabolic function of the gut microbiota is disrupted under obese conditions, which aligns with the *E. faecium*‐mediated regulatory mechanism of LEA synthesis.

### LEA improved metabolic phenotypes and regulated mitochondrial oxidative phosphorylation pathway in obese mice

**FIGURE 5:**
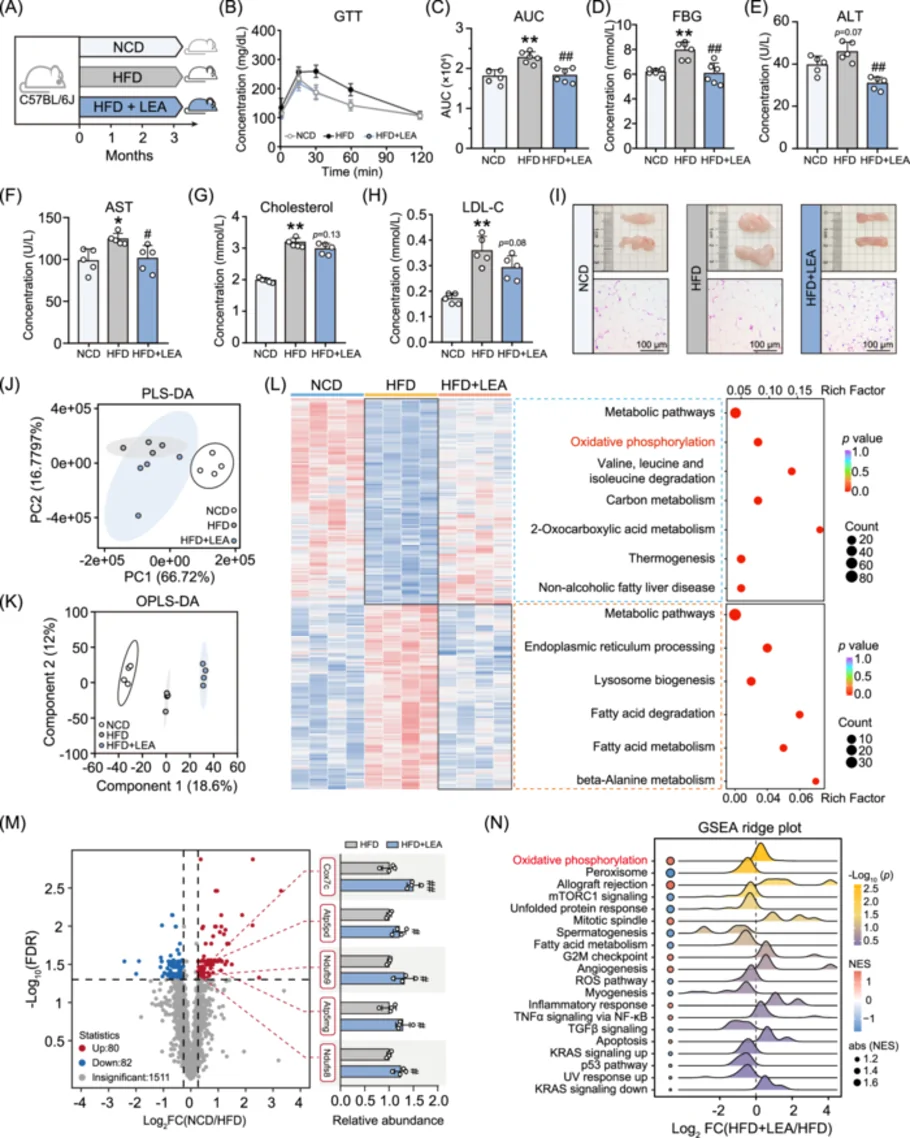
LEA alleviated obesity and activated oxidative phosphorylation in liver tissues. (A) Schematic diagram. (B) Glucose tolerance test (GTT) curves. (C) AUC of GTT. (D) Concentration of FBG. (E–F) Concentrations of alanine transaminase (ALT) and aspartate transaminase (AST). (G, H) Concentrations of cholesterol and LDL‐C. (I) H&E staining images of epididymal adipose tissues. Scale bar = 100 µm. (J–K) Score plots of PLS‐DA and orthogonal partial least squares discriminant analysis (OPLS‐DA) based on hepatic proteomic profiles. (L) Heatmap of differentially expressed proteins (left) and KEGG enrichment bubble plots (right). (M) Volcano plots illustrating differential protein expression changes. (N) Gene set enrichment analysis (GSEA) ridge plot of oxidative phosphorylation pathway. Data were expressed as mean ± SD (*n* = 4–6). *p < 0.05, **p < 0.01 versus NCD group; ^#^ *p* < 0.05, ^##^ *p* < 0.01 versus HFD group. FBG, fasting blood glucose; LEA, leucic acid.

To explore the ameliorative effect of LEA on metabolic phenotypes and its underlying molecular mechanism in HFD‐induced obese mice, an HFD‐induced obese model was established in C57BL/6J mice, which were subsequently treated with LEA for 3 months. The experimental design was shown in Figure 5A. The body weight of mice in the HFD group was 6.10% higher than that of the NCD group. Compared with the HFD group, LEA intervention effectively inhibited weight gain in mice (Figure S6A,B). White adipose tissue (WAT) weight was markedly elevated in the HFD group compared with the NCD group, and LEA intervention significantly attenuated WAT accumulation in obese mice (Figure S6C). Glucose phenotypic analysis revealed that compared with the NCD group, HFD mice exhibited obvious glucose intolerance, accompanied by increased area under the curve (AUC) of the glucose tolerance test (GTT) and elevated fasting blood glucose (FBG) levels. LEA intervention markedly reversed these abnormalities and effectively ameliorated glucose metabolic disorders in mice (Figure 5B–D). Plasma biochemical detection showed that LEA treatment reduced the plasma levels of alanine transaminase (ALT), aspartate transaminase (AST), total cholesterol, and LDL‐C in HFD mice, thereby alleviating HFD‐induced liver injury and lipid metabolic dysfunction (Figure 5E–H). Staining results of liver and epididymal adipose tissues showed that mice in the NCD group presented normal liver structure without obvious lipid droplet deposition (Figure 5I and Figure S6D). In contrast, HFD mice displayed enlarged and yellowish liver as well as increased epididymal adipose tissue. LEA intervention significantly relieved the above pathological damage and reduced lipid droplet accumulation.

Further proteomic analysis revealed the regulatory effects of LEA. Partial least squares discriminant analysis (PLS‐DA) and OPLS‐DA showed obvious separation among the NCD group, HFD group, and HFD + LEA group in the principal component space, indicating significant differences in hepatic protein expression profiles among the three groups (Figure 5J,K). LEA effectively remodeled the abnormal protein expression profile induced by HFD. Differential protein enrichment analysis demonstrated that the differential proteins in the HFD group compared with the NCD group were mainly enriched in pathways including oxidative phosphorylation, metabolic pathways, and valine/leucine/isoleucine degradation (Figure 5L). LEA intervention markedly reversed the abnormal enrichment trend of these pathways, and mainly regulated key pathways such as oxidative phosphorylation and fatty acid degradation. Gene set enrichment analysis (GSEA) and differential protein volcano plot further verified the pathway regulation results. Volcano plot analysis showed that the HFD group significantly downregulated 80 proteins, including core oxidative phosphorylation proteins such as Cox7c, Atp5pd, Ndufb9, Atp5mg, and Ndufs8. Further analysis revealed that LEA intervention restored the expression levels of these proteins (Figure 5M). GSEA ridge plot showed that the mitochondrial oxidative phosphorylation pathway was significantly enriched in the HFD + LEA group (Figure 5N).

To investigate whether LEA is a key effector molecule mediating the anti‐obesity effects of *E. faecium*, we also examined the regulatory role of LEA in lipid synthesis and mitochondrial oxidative phosphorylation. The results demonstrated that LEA intervention markedly increased the activity of mitochondrial complex I and promoted ATP production in HFD‐induced obese mice (Figure S6E,F). LEA similarly suppressed lipogenesis by promoting the phosphorylation of AMPK and ACC, while downregulating SREBP1 expression (Figure S6G,H).

### LEA alleviated PA‐induced lipid metabolism disorder in AML12 cells by improving mitochondrial oxidative phosphorylation

**FIGURE 6:**
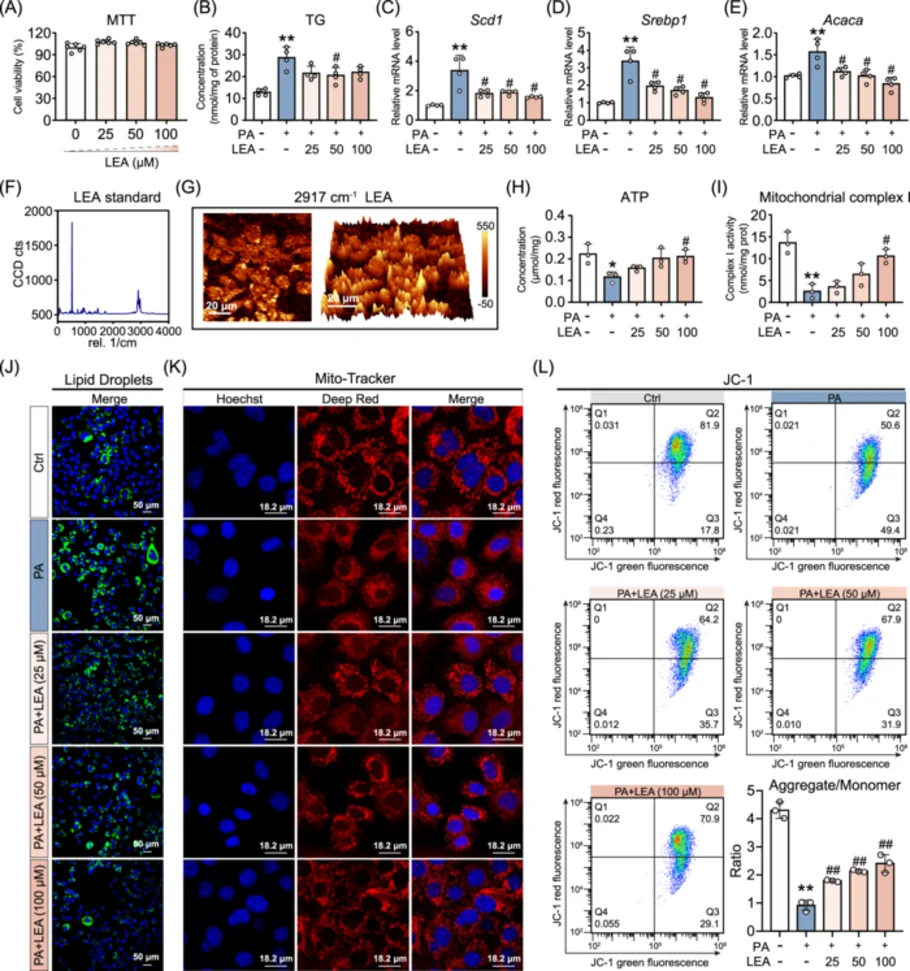
LEA ameliorated palmitic acid (PA)‐induced lipid metabolism disorder and mitochondrial dysfunction in AML12 hepatocytes. (A) Cell viability of AML12 cells treated with different concentrations of LEA (0–100 μM) assessed by MTT assay. (B) Intracellular TG content in AML12 cells with or without PA and LEA intervention. (C–E) Relative mRNA expression levels of key lipogenic genes *Scd1*, *Srebp1*, and *Acaca*. (F) Raman spectrum of LEA standard. (G) Raman imaging of LEA‐treated AML12 cells visualized at the 2917 cm^−1^ characteristic peak. (H) Intracellular ATP content. (I) Activity of mitochondrial respiratory chain complex I. (J) Representative images of lipid droplet staining (green) in AML12 cells. Scale bar = 50 μm. (K) Representative images of Mito‐Tracker staining (red, mitochondria) and Hoechst staining (blue, nuclei). Scale bar = 18.2 μm. (L) Detection and relative quantitative analysis of mitochondrial membrane potential in AML12 cells by flow cytometry (red: JC‐1 aggregate, green: JC‐1 monomer). Data were expressed as mean ± SD (*n* = 3–6). *p < 0.05, **p < 0.01 versus Ctrl group; ^#^ *p* < 0.05, ^##^ *p* < 0.01 versus PA group.

MTT cell viability assay verified that LEA exhibited no obvious cytotoxicity against AML12 cells within the concentration range of 0–100 μmol/L, and this concentration gradient was therefore adopted for subsequent intervention experiments (Figure 6A). PA exposure markedly induced lipid metabolism disorder in AML12 cells. LEA treatment effectively alleviated the elevation of intracellular TG content and massive lipid droplet accumulation, and regulated the mRNA expression levels of key lipogenic genes, including *Scd1*, *Srebp1*, and *Acaca*, confirming the favorable efficacy of LEA in ameliorating PA‐triggered hepatic lipid metabolism disturbance (Figure 6B–E,J). Raman spectroscopy and Raman imaging detected specific signals at the characteristic absorption peak of 2917 cm^−1^, demonstrating that LEA could successfully penetrate into AML12 cells and exert pharmacological effects (Figure 6F–G). Consistent with the findings of in vivo experiments, LEA could repair mitochondrial oxidative phosphorylation damage secondary to lipid metabolic imbalance. PA treatment suppressed cellular ATP synthesis and sharply reduced the activity of mitochondrial respiratory chain complex I, while LEA concentration‐dependently restored ATP content and complex I activity, thereby alleviating oxidative phosphorylation dysfunction (Figure 6H–I). Detection of mitochondrial structure and function revealed that PA disrupted mitochondrial membrane potential homeostasis and decreased the JC‐1 red/green fluorescence ratio, accompanied by mitochondrial fragmentation and impaired network structure. LEA intervention reversed the above pathological alterations, recovered mitochondrial membrane potential, and restored the integrity of mitochondrial network architecture (Figure 6K–L). In summary, LEA could enter hepatocytes, restore respiratory chain complex activity, elevate ATP synthesis efficiency, stabilize membrane potential, and maintain normal mitochondrial morphology. These effects corrected abnormal oxidative phosphorylation and energy metabolism disorders, further diminished excessive intracellular lipid deposition, and ultimately improved PA‐induced lipid metabolism disorder in hepatocytes.

### High‐content screening for selecting dietary fiber for enriching *E. faecium*

**FIGURE 7:**
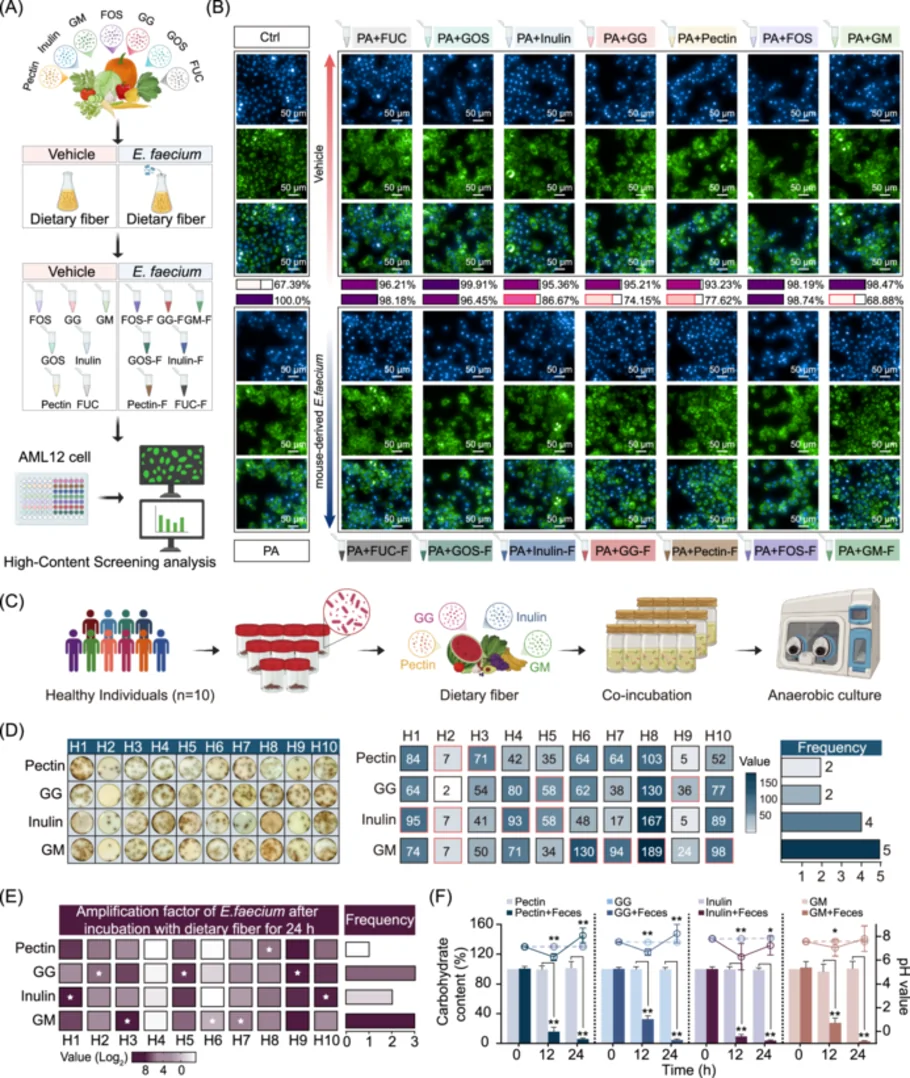
High‐content screening system identified the optimal dietary fiber for enriching mouse‐derived *E. faecium*. (A) Experimental scheme. (B) High‐content cellular imaging analysis of lipid droplet fluorescent staining in AML12 cells, including fluorescence images and quantitative heatmap of lipid droplet area. (C) Schematic diagram. (D) Image of *E. faecium* on Enterococcus screening plate and its counting heatmap. (E) Multiplication factor of *E. faecium* after incubation of dietary fibers with human feces for 24 h. (F) Changes in total carbohydrates and pH of fermentation supernatants after 24 h incubation of dietary fibers with human feces. The bar graph represents the change in carbohydrate content, and the line graph represents the change in pH value. Scale bar = 50 µm. Data were expressed as mean ± SD. *p < 0.05, **p < 0.01. FOS, Fructo‑oligosaccharide; FOS‐F, FOS fermentation broth; FUC, Fucoidan; FUC‐F, FUC fermentation broth; GG, Guar gum; GG‐F, GG fermentation broth; GM, Glucomannan; GM‐F, GM fermentation broth; GOS, Galacto‑oligosaccharide; GOS‐F, GOS fermentation broth; inulin‐F, inulin fermentation broth; pectin‐F, pectin fermentation broth.

To avoid the uncertainty of live bacterial therapy, we introduced dietary fibers as a medium to enhance the probiotic activity and abundance of *E. faecium* in the host. We selected multiple dietary fibers with prebiotic activity, including pectin, inulin, GM, Fructo‑oligosaccharide (FOS), Guar Gum (GG), Galacto‑oligosaccharide (GOS), and Fucoidan (FUC). These dietary fibers were individually co‐fermented with *E. faecium* (*Ef*‐M and *Ef*‐H) in vitro, and the resulting fermentation supernatants were applied to AML12 cells with lipid accumulation. The cells were then evaluated using a high‐content screening system (Figure 7A). The results showed that the total lipid droplet area per cell in the PA group was significantly larger than that in the Ctrl group. Compared with the PA group, dietary fiber solutions alone failed to reduce the lipid droplet area in AML12 cells, while GM fermentation broth (GM‐F), pectin fermentation broth (pectin‐F), GG fermentation broth (GG‐F), and inulin fermentation broth (inulin‐F) groups significantly reduced the total lipid droplet area per cell (Figure 7B and Figure S7A). To assess the enrichment of *E. faecium* by these four dietary fibers, we conducted an in vitro fermentation assay using each fiber with 10 human fecal samples (Figure 7C). Subsequently, we spread the fermented bacteria onto an Enterococcus screening plate to observe its abundance. As shown in the counting results of Figure 7D–E, the ability of the four dietary fibers to enrich *Enterococcus* differed. Among them, GM showed superior enrichment of *E. faecium* in most donors, especially H2, H6, H7, H8, and H10, with variable effects in other individuals. Meanwhile, we used qPCR to determine the abundance of *E. faecium*. The heatmap showed the multiplication factor of *E. faecium* after incubation with different dietary fibers for 24 h. GG and GM performed better. Moreover, total carbohydrate and pH changes were examined in the fermentation supernatant. The human gut microbiota could utilize all four dietary fibers (Figure 7F and Figure S7B). In conclusion, GM is the optimal dietary fiber medium for enriching *E. faecium* in the host.

### Dietary fiber GM‐enriched *E. faecium* and ameliorated HFD‐induced obesity

To determine the enrichment effect of GM on *E. faecium* in vivo and its anti‐obesity activity, we used GM to intervene in obese mice (Figure S8A). As shown in Figure S9A,B, the body weight of HFD‐fed mice was 30% higher than that of the NCD group, and GM intervention effectively attenuated weight gain in obese mice. GM supplementation alleviated glucose intolerance in HFD‐fed mice (Figure S8B,C and Figure S9C). Meanwhile, GM effectively reduced the weight of WAT (subcutaneous adipose, mesenteric adipose, and epididymal adipose) and ameliorated dyslipidemia by lowering cholesterol, TG, and LDL‐C in HFD‐fed mice (Figure S8D,E). H&E and Oil Red O staining of mesenteric adipose and liver demonstrated that GM treatment suppressed lipid accumulation in obese mice (Figure S8F). The mRNA expression of lipogenic genes including *Cebp1*, *Fasn*, and *Srebp1* was inhibited by GM intervention, while *Pparα* expression was upregulated (Figure S9D). Subsequently, we use 16S rDNA sequencing to evaluate the effect of GM on the gut microbiota of obese mice. As displayed in Figure S8G,H, GM intervention significantly increased the α‐diversity (Shannon and Simpson indices) of gut microbiota in HFD mice. The gut microbiota structure in HFD mice was also remodeled by GM (Figure S8I and Figure S9E). Similar to in vitro experiments, GM also increased the abundance of *E. faecium* in vivo (Figure S8J–L).

Then, we depleted gut microbiota in obese mice to verify if GM's anti‐obesity effect correlates with *E. faecium* enrichment (Figure S10A). The results showed no statistical difference in body weight, FBG, cholesterol, TG, and LDL‐C between HFD + antibiotic mixture (ABx) and HFD + ABx + GM groups (Figure S10B–F). Meanwhile, 16S rDNA gene sequencing results indicated that ABx significantly reduced the α‐diversity, altered the microbiota structure, and decreased the abundance of bacteria, including *Enterococcus*, in HFD‐fed mice. GM did not restore the damage caused by ABx to the gut microbiota of mice (Figure S10G–L).

## DISCUSSION

Obesity is a multifactorial disease driven by the interaction of genetic and environmental factors, with high‐fat diets and lifestyle changes contributing to its high prevalence [34]. Gut microbiota is an essential adaptive result of environmental factors [35], and targeted modulation of the gut microbiota has emerged as a novel strategy for obesity intervention [36]. Therefore, it is crucial to identify potential probiotics and elucidate the molecular mechanisms. Through dual‐cohort screening and independent validation, we found that the abundance of *E. faecium* was significantly reduced in obese subjects. Functional studies showed that this bacterium converted leucine into LEA via putative BCAAs metabolic enzymes, thereby alleviating obesity by regulating mitochondrial oxidative phosphorylation. Combined with high‐content screening and an in vitro fermentation model, we confirmed that the dietary fiber GM exerted anti‐obesity effects by promoting the proliferation of *E. faecium*. These findings reveal the critical role of the *E. faecium*‐LEA axis in obesity intervention and provide new insights into precision dietary intervention against obesity.

*E. faecium* is a core commensal gram‐positive bacterium in the intestinal tract of humans and animals [37]. In the study, we isolated two strains of *E. faecium* from human and mouse feces (Figure 1). The strain has been reported to secrete bacteriostatic substances that inhibit oral pathogen *Streptococcus mutans* UKMCC 1019 [38], and child intestinal‐derived *E. faecium* exhibits cholesterol‐lowering capacity [39], which is consistent with our results (Figure 2). Although *E. faecium SF68* has been recognized as a safe feed additive, antibiotic resistance restricts its broader application [40]. Currently, the beneficial functions of *E. faecium* and its metabolites have been reappraised. *E. faecium* secretes SagA to activate host immunity and enhance defense against pathogenic infection [41]. *E. faecium* and its extracellular polysaccharides synergize with sorafenib to ameliorate hepatocellular carcinoma in mice by modulating tumor immunity [42]. Furthermore, *E. faecium* also protects hosts against *Salmonella* invasion and reduces bacterial infection risk [43, 44]. Our study confirmed that both human‐ and mouse‐derived *E. faecium* effectively ameliorate lipid metabolism disorders in obese mice without causing adverse reactions such as diarrhea (Figure 2). Whole‐genome sequencing safety assessment indicated that the resistance genes of the isolated strains are chromosomally encoded rather than plasmid‐borne, leading to a low risk of horizontal gene transfer. Moreover, these strains exhibit distinct genetic differences from clinically prevalent vancomycin‐resistant *E. faecium*. Given the potential risks of live bacteria intervention and the loss of anti‐obesity efficacy of inactivated *E. faecium*, we adopted a prebiotic‐based indirect enrichment strategy. Dietary fiber supplementation specifically promotes the proliferation of endogenous *E. faecium* in vivo. This strategy ensures anti‐obesity efficacy while avoiding the potential hazards of exogenous resistant strains, providing a safe and feasible strategy for microbiota‐based intervention of metabolic diseases.

The benefits of dietary fiber on metabolism and obesity have been widely reported [45]. In the study, in vitro anaerobic cultivation confirmed that GM, pectin, GG and inulin can all be degraded and utilized by the human gut microbiota (Figure 7). Restricted by the sample size of 10 donors, the enrichment of *E. faecium* by GM varied among individuals. Nevertheless, the abundance of *E. faecium* increased in all samples, and interindividual differences in population responses may be associated with baseline microbiota composition. In vivo experiments combined with literature comparison indicate that the enrichment of *E. faecium* by GM is dose‐ and molecular weight‐dependent, which may explain the inconsistent findings in previous relevant studies [46, 47, 48, 49]. *E. faecium* harbors complete glycosidase‐encoding genes and can utilize raffinose and lactulose [50, 51, 52]. Different intestinal strains have different polysaccharide utilization systems, which determines that gut microbiota can be precisely regulated by dietary fiber and generate various active substances, thus participating in the life activities of the host [13]. Previous studies have demonstrated that under a high‐fat diet, *Fusimonas intestini* promotes the synthesis of long‐chain fatty acids and aggravates obesity [53], whereas *Parabacteroides johnsonii* alleviates metabolic disorders by degrading BCAAs [20]. These findings suggest that microbial metabolites are pivotal to deciphering microbiota‐host interactions. This study reveals that heat‐killed *E. faecium* fails to ameliorate dyslipidemia and fat accumulation in obese mice (Figure 2), demonstrating that the anti‐obesity activity of this bacterium relies not on its structural components but on metabolites. Through a combination of cellular functional assays and metabolomic analysis, we identified this key bioactive substance as LEA and provided confirmation of *E. faecium*'s biological capacity to synthesize LEA (Figure 4). LEA generation depends on a series of enzymatic reactions within *E. faecium* that metabolize leucine. Heat treatment inactivates intracellular enzymes and blocks LEA biosynthesis, which constitutes the fundamental reason for the loss of activity of heat‐killed bacteria. As a leucine derivative originally isolated from *Lactobacillus plantarum*, LEA has been reported to inhibit periodontal pathogens [54, 55] and improve metabolic dysfunction associated with fatty liver disease [56]. However, the potential mechanisms by which LEA alleviates obesity remain to be elucidated.

Mitochondrial oxidative phosphorylation dysfunction is a core component of obesity‐related metabolic disorders [57]. The present study demonstrated that a high‐fat diet suppresses mitochondrial complex I activity and ATP synthesis in the mouse liver. As a leucine metabolite derived from *E. faecium*, LEA restored oxidative phosphorylation function and repaired mitochondrial integrity (Figure 6). Based on existing reports, potential mechanisms by which LEA restores complex I activity include activation of the HCAR2‐AMPK pathway, which subsequently promotes mitochondrial biogenesis and oxidative phosphorylation, or indirect protection of complex I from oxidative stress‐induced injury by alleviating lipid peroxidation and ferroptosis‐related damage [58]. These hypotheses require further experimental validation using targeted interventions against key signaling molecules.

This study has certain limitations. As a retrospective cohort study, it failed to systematically collect data on participants' dietary intake and hypoglycemic and lipid‐lowering medication use using standardized tools. Second, we focused solely on LEA as a single metabolite and did not explore whether other active metabolites produced by *E. faecium* (such as short‐chain fatty acids) synergistically contribute to the anti‐obesity effects. We were unable to detect LEA levels in the feces, plasma, or liver following *E. faecium* colonization, which precluded a correlation analysis between *E. faecium* abundance and in vivo LEA concentrations. Furthermore, only two *E. faecium* strains from specific sources were validated, and it remains unclear whether strains with different origins and genetic backgrounds possess consistent LEA‐producing capacity and anti‐obesity efficacy. We hypothesize that *E. faecium* synthesizes LEA via BCAAs metabolic enzymes, yet this hypothesis lacks direct evidence from genetic manipulation experiments, such as gene knockout. Future studies should combine techniques including gene editing, gnotobiotic animal models, and metabolic flux tracing to elucidate the LEA synthesis pathway and assess the generalizability across strains, thereby providing a more complete theoretical basis for precision interventions targeting this axis.

## CONCLUSION

In summary, through fecal metagenomic data mining combined with clinical cohort validation, this study identified a gut bacterial strain with anti‐obesity potential, *E. faecium*. Animal experiments demonstrated that colonization with *E. faecium* alleviated HFD‐induced obesity. Mechanistically, *E. faecium* utilizes putative BCAAs metabolic enzymes to convert leucine into LEA, and exogenous LEA supplementation also regulate mitochondrial oxidative phosphorylation and improve energy metabolism. Furthermore, using high‐content screening in conjunction with an in vitro fermentation model, we found that the dietary fiber GM effectively enriched *E. faecium*, thereby exerting anti‐obesity effects. Collectively, this study reveals the critical role of the *E. faecium*‐LEA axis in regulating host energy metabolism and provides a theoretical foundation for precision dietary fiber intervention strategies targeting the gut microbiota.

## METHODS

### Selecting and culturing *E. faecium*

Healthy human feces were diluted 1:20 in sterile anaerobic PBS, filtered, and inoculated into Enterococcus broth (HB0133‐2, Hopebio) for two anaerobic passages. Serial dilutions (10^−6^ to 10^−8^) were plated on Enterococcus agar (HB0133, Hopebio, China), and brown colonies were isolated and subcultured twice. Single colonies were selected for whole‐genome sequencing on the Oxford Nanopore platform. Two *E. faecium* strains from human and mouse feces were cultured in BHI (HB8297‐1, Hopebio), preserved in 20% glycerol at −80°C, and subcultured from BHI agar to broth as needed. All human procedures complied with the Declaration of Helsinki, with written informed consent obtained.

### Animal studies

Male C57BL/6J mice (6 weeks old) were purchased from Hubei Center for Disease Control and Prevention, housed in IVC systems (20 ± 2°C, 12 h light/dark cycle), acclimatized for 1 week, and euthanized with isoflurane post‐experiment.

Two *Enterococcus faecium* strains were used in this study: a human‐derived strain (*Ef*‐H) and a mouse‐derived strain (*Ef‐*M). For animal experiments, L*Ef*‐H and L*Ef*‐M refer to live human‐derived and mouse‐derived *E. faecium*, respectively; K*Ef*‐H and K*Ef*‐M refer to heat‐killed human‐derived and mouse‐derived *E. faecium*, respectively.

To investigate *E. faecium* in HFD‐induced obesity, male C57BL/6J mice were fed NCD or HFD (KEAO XIELI) for 3 months and divided into six groups: NCD, HFD, HFD+L*Ef*‐M, HFD+K*Ef*‐M, HFD+L*Ef*‐H, and HFD+K*Ef*‐H. *E. faecium* was cultured anaerobically in BHI at 37°C. Live bacteria (log phase) were resuspended in sterile anaerobic PBS and gavage (1 × 10^9^ colony‐forming unit (CFU)/0.2 mL/d) to HFD+L*Ef*‐M group and HFD+L*Ef*‐H group. Heat‐killed *E. faecium* (121°C, 30 min) was similarly prepared and administered to HFD+K*Ef*‐M group and HFD+K*Ef*‐H group. NCD and HFD groups received sterile PBS. After 3 months, mice were sacrificed with isoflurane (792632, Merck). Liver and epididymal fat were either fixed in formalin (G1101, Servicebio) for histology or snap‐frozen in liquid nitrogen and stored at −80°C.

To investigate the function of LEA (H302104, Aladdin) on the protective effect of HFD‐induced obesity mice, male C57BL/6J mice were divided randomly into three groups after adaptive feeding: NCD group, HFD group, HFD + LEA group. During 3 months, mice were given NCD or HFD, and mice in the HFD + LEA group received 200 mg/kg LEA solution by drinking water daily.

To assay the protective effects of GM on obese mice, male C57BL/6J mice were randomized into four groups: NCD group, HFD group, NCD + GM group, and HFD + GM group. Mice were given NCD or HFD for 3 months, and 1 mg/mL GM (dissolved in drinking water) was used for treatment.

### In vitro co‐incubation of human feces with dietary fibers

Feces from 10 healthy individuals were collected separately and washed with sterile anaerobic PBS to remove large particulate matter, and then resuspended into a 10% fecal suspension. We used inulin, pectin, GG, and GM to co‐incubate with each of the 10 fecal suspensions. Dietary fibers were dissolved in the BHI medium at a final concentration of 2.5 mg/mL. After autoclaving, fecal suspension was added to the above medium at a rate of 5% and incubated anaerobically at 37°C. Samples were collected at 0 h, 12 h, and 24 h for the determination of total carbohydrates, pH, and subsequent extraction of fecal DNA.

### Histological analysis

H&E staining was performed on liver and epididymal adipose tissue fixed with paraformaldehyde using an H&E staining kit (C0105, Beyotime). The detailed Oil Red O staining steps were described in the Supplementary Methods.

### Biochemical analysis

The levels of cholesterol, TG, LDL‐C, AST, and ALT in plasma were assayed by commercial biochemical analyzer kits (Vet‐10, Vet‐11, Vet‐26, Vet‐02, and Vet‐01, Mindray). Determination of TG content in AML12 cells was determined using a cell TG assay kit (E1013, Applygen Technologies).

### RNA extraction and qPCR analysis

RNA of liver tissue and AML12 cells was extracted using trizol (NR001, zhhcbio), and then RNA was reverse‐transcribed by a cDNA synthesis kit (AM1810, ALLMEEK). The primers used in the experiment are listed in Table S4. The expression of target genes was normalized against that of *gapdh*, and fold changes were calculated using a 2^(^^−ΔΔCT)^ method.

### Statistical analysis

Results were presented as mean ± SD. When conducting statistical difference analysis between groups, we first tested the normality and homogeneity of variance of the data using the Shapiro–Wilk test and Brown–Forsythe test. If the data conformed to both normal distribution and homogeneity of variance simultaneously, we performed the analysis using one‐way analysis of variance (ANOVA) combined with the Tukey–Kramer test. If the data did not conform to the normal distribution, we used the Kruskal–Wallis *H* test combined with Dunn's test for post‐hoc multiple comparisons. Data were calculated using Prism version 8.0.1, GraphPad Software.

For proteomic analysis, all samples were uniformly preprocessed and subjected to mass spectrometry analysis within the same batch. Protein identification and quantification were performed using Spectronaut 16.0. Proteins with missing quantitative signals in more than 50% of samples were excluded, and the remaining missing values were imputed using the k‐nearest neighbor algorithm. Pooled quality control samples were interspersed throughout the run to monitor instrument stability. The coefficient of variation (CV) for each protein was calculated based on the QC samples, and proteins with a CV > 30% were excluded. A PLS‐DA model was constructed to evaluate group separation, and its robustness was assessed using sevenfold cross‐validation combined with 200 permutation tests to prevent overfitting. *p*‐values from multiple comparisons were corrected using the Benjamini–Hochberg (BH) method. DEPs were screened using a threshold of FDR < 0.05 and absolute fold change > 1.2.

For repeated‐measures data (e.g., body weight changes, GTT curves, and in vitro fermentation), a two‐way repeated‐measures ANOVA (group × time) was used to determine statistical significance, followed by Tukey's post hoc test for multiple comparisons (*p* < 0.05).

For microbiome analysis, alpha diversity indices (Richness, Shannon, and Simpson) were calculated using the *picante* package (v1.8). Beta diversity was assessed by Bray–Curtis dissimilarity matrices with 999 permutations using the *vegan* package (v2.6‐4). Correlation networks were constructed using Spearman's rank correlation coefficient (|r| > 0.6, *p* < 0.05) and visualized in Gephi. Taxonomic differences among groups were identified using LEfSe analysis, with a linear discriminant analysis score threshold of 3.5. Intergroup differences in species abundance were analyzed using the *t*‐test, with the Wilcoxon rank‐sum test applied when parametric assumptions were violated. *p*‐values were adjusted for multiple comparisons using the BH method.

Metabolomics data were preprocessed and statistically analyzed using the MetaboAnalyst online platform. Missing values for metabolites with a missing rate of less than 50% were imputed using the *k*‐nearest neighbor algorithm. All subsequent result charts were generated using the MetWare metabolomics cloud platform. For high‐content screening, quantitative image analysis was conducted using the RMS Lipid Droplet Analysis module embedded in the Harmony instrument software.

## AUTHOR CONTRIBUTIONS

**Baifei Hu**: Validation; visualization; writing—original draft. **Qianru Gao**: Conceptualization; data curation; formal analysis. **Junmin Hui**: Validation; visualization. **Mengqiong Wang**: Conceptualization; data curation. **Haiyan Gao**: Project administration. **Kai Li**: Resources; software; supervision. **Junping Zheng**: Conceptualization. **Zhe Dai**: Methodology; investigation. **Songlin Liu**: Funding acquisition. **Hongtao Liu**: Writing—review and editing; funding acquisition.

## CONFLICT OF INTEREST STATEMENT

The authors declare no conflicts of interest.

## ETHICS STATEMENT

All experiments involving animals were conducted according to the ethical policies and procedures approved by the Animal Ethical Experimentation Committee of the Hubei University of Chinese Medicine (NO. SYXK 2023‐0067). The experiments were in compliance with the ARRIVE guidelines and carried out in accordance with the U.K. Animals (Scientific Procedures) Act, 1986 and associated guidelines, EU Directive 2010/63/EU. The study involving human participants was approved by the Ethics Committee of the Hubei University of Chinese Medicine, with the approval number 2024020, and carried out in accordance with The Code of Ethics of the World Medical Association (Declaration of Helsinki). Informed consent has been obtained from the participants in the experiment.

## Supporting information


**Figure S1:** Analysis of gut microbiota diversity and differential species between healthy and obese groups.
**Figure S2:** Genomic characteristics and functional annotation of the two isolated *E. faecium* strains.
**Figure S3:** Validation of strain‐specific detection and metabolic effects following *E. faecium* colonization.
**Figure S4:** *E. faecium* intervention restored hepatic mitochondrial energy metabolism in HFD‐fed mice.
**Figure S5:** LEA was a candidate bioactive metabolite produced by *E. faecium*.
**Figure S6:** LEA alleviated hepatic pathological damage and improved mitochondrial function in HFD‐fed mice.
**Figure S7:** High‐content screening of AML12 cells and TLC analysis of fermentation supernatants.
**Figure S8:** GM improved HFD‐induced obesity in mice and enriched *E.faecium* in vivo.
**Figure S9:** GM ameliorated metabolic disorders in obese mice.
**Figure S10:** Alleviation of obesity by GM was microbiota‐dependent.

**Table S1:** Obesity‐associated species (2017_ Liu cohort (PRJEB12123)).
**Table S2:** Species‐level microbial taxa positively correlated with dietary fiber intake (2024_ Wang cohort (QIITA, ID 11666)).
**Table S3:** Genomic features of *E. faecium*.
**Table S4:** Primer sequence.

## Data Availability

The data that support the findings of this study are available from the corresponding author upon reasonable request. The mass spectrometry proteomics data have been deposited to the ProteomeXchange Consortium (https://proteomecentral.proteomexchange.org) via the iProX partner repository with the dataset identifier PXD082421 (https://www.iprox.cn/page/MSV022.html). Raw metagenomic sequencing data have been deposited in the GSA‐Human database (accession number: HRA020257), which are publicly accessible at https://ngdc.cncb.ac.cn/gsa-human/submit/hra/subHRA031992/finishedOverview. Raw whole‐genome sequencing data of the two *E. faecium* strains have been deposited in the Genome Sequence Archive (GSA: CRA048052) in the National Genomics Data Center that are publicly accessible at https://ngdc.cncb.ac.cn/gsub/submit/bioproject/subPRO103622/overview. Raw 16S rDNA sequencing data have been deposited in the NCBI Sequence Read Archive (SRA) under BioProject accession number PRJNA1192758 and are publicly available (https://submit.ncbi.nlm.nih.gov/subs/sra/SUB14901088/overview). The data and scripts used are saved in GitHub https://github.com/qianrugao/Figure-data-2026-567. Supplementary materials (figures, tables, graphical abstract, slides, videos, Chinese translated version, and updated materials) may be found in the online DOI or iMeta Science http://www.imeta.science/.

## References

[1] Piché, Marie‐Eve , André Tchernof , and Jean‐Pierre Després . 2020. “Obesity Phenotypes, Diabetes, and Cardiovascular Diseases.” Circulation Research 126: 1477–1500. 10.1161/circresaha.120.316101 32437302

[2] Lister, Natalie B. , Louise A. Baur , Janine F. Felix , Andrew J. Hill , Claude Marcus , Thomas Reinehr , Carolyn Summerbell , and Martin Wabitsch . 2023. “Child and Adolescent Obesity.” Nature Reviews Disease Primers 9: 24. 10.1038/s41572-023-00435-4 37202378

[3] Kerr, Jessica A. , George C. Patton , Karly I. Cini , Yohannes Habtegiorgis Abate , Nasir Abbas , Abdallah H. A. Abd Al Magied , Samar Abd ElHafeez , et al. 2025. “Global, Regional, and National Prevalence of Child and Adolescent Overweight and Obesity, 1990–2021, With Forecasts to 2050: A Forecasting Study for the Global Burden of Disease Study 2021.” The Lancet 405: 785–812. 10.1016/s0140-6736(25)00397-6 PMC11920006 40049185

[4] Ng, Marie , Emmanuela Gakidou , Justin Lo , Yohannes Habtegiorgis Abate , Cristiana Abbafati , Nasir Abbas , Mohammadreza Abbasian , et al. 2025. “Global, Regional, and National Prevalence of Adult Overweight and Obesity, 1990–2021, With Forecasts to 2050: A Forecasting Study for the Global Burden of Disease Study 2021.” The Lancet 405: 813–838. 10.1016/s0140-6736(25)00355-1 PMC11920007 40049186

[5] Anazco, Diego , and Andres Acosta . 2024. “Precision Medicine for Obesity: Current Evidence and Insights for Personalization of Obesity Pharmacotherapy.” International Journal of Obesity 49: 452–463. 10.1038/s41366-024-01599-z PMC11931505 39127792

[6] Ward, Zachary J. , Sara N. Bleich , Angie L. Cradock , Jessica L. Barrett , Catherine M. Giles , Chasmine Flax , Michael W. Long , and Steven L. Gortmaker . 2019. “Projected U.S. State‐Level Prevalence of Adult Obesity and Severe Obesity.” New England Journal of Medicine 381: 2440–2450. 10.1056/NEJMsa1909301 31851800

[7] González‐Muniesa, Pedro , Miguel‐Angel Mártinez‐González , Frank B. Hu , Jean‐Pierre Després , Yuji Matsuzawa , Ruth J. F. Loos , Luis A. Moreno , George A. Bray , and J. Alfredo Martinez . 2017. “Obesity.” Nature Reviews. Disease Primers 3: 17034. 10.1038/nrdp.2017.34 28617414

[8] Berthoud, Hans‐Rudolf , Christopher D. Morrison , and Heike Münzberg . 2020. “The Obesity Epidemic in the Face of Homeostatic Body Weight Regulation: What Went Wrong and How Can It Be Fixed?” Physiology & Behaviour 222: 112959. 10.1016/j.physbeh.2020.112959 PMC7321883 32422162

[9] Kivimäki, Mika , Eeva Kuosma , Jane E. Ferrie , Ritva Luukkonen , Solja T. Nyberg , Lars Alfredsson , G David Batty , et al. 2017. “Overweight, Obesity, and Risk of Cardiometabolic Multimorbidity: Pooled Analysis of Individual‐Level Data for 120 813 Adults From 16 Cohort Studies From the USA and Europe.” The Lancet. Public health 2: e277–e285. 10.1016/s2468-2667(17)30074-9 PMC5463032 28626830

[10] Ursell, Luke K. , Henry J. Haiser , Will Van Treuren , Neha Garg , Lavanya Reddivari , Jairam Vanamala , Pieter C. Dorrestein , Peter J. Turnbaugh , and Rob Knight . 2014. “The Intestinal Metabolome: An Intersection Between Microbiota and Host.” Gastroenterology 146: 1470–1476. 10.1053/j.gastro.2014.03.001 PMC4102302 24631493

[11] Cani, Patrice D. , and Matthias Van Hul . 2024. “Gut Microbiota in Overweight and Obesity: Crosstalk With Adipose Tissue.” Nature Reviews. Gastroenterology & Hepatology 21: 164–183. 10.1038/s41575-023-00867-z 38066102

[12] Torres‐Fuentes, Cristina , Harriët Schellekens , Timothy G. Dinan , and John F. Cryan . 2017. “The Microbiota‐Gut‐Brain Axis in Obesity.” The Lancet. Gastroenterology & Hepatology 2: 747–756. 10.1016/s2468-1253(17)30147-4 28844808

[13] Liu, BingNan , XiaoTong Liu , ZiHan Liang , and JiHui Wang . 2021. “Gut Microbiota in Obesity.” World Journal of Gastroenterology 27: 3837–3850. 10.3748/wjg.v27.i25.3837 PMC8291023 34321848

[14] Sheykhsaran, Elham , Amin Abbasi , Hamed Ebrahimzadeh Leylabadlo , Javid Sadeghi , Samaneh Mehri , Fariba Naeimi Mazraeh , Hadi Feizi , and Hossein Bannazadeh Baghi . 2023. “Gut Microbiota and Obesity: An Overview of Microbiota to Microbial‐Based Therapies.” Postgraduate Medical Journal 99: 384–402. 10.1136/postgradmedj-2021-141311 37294712

[15] Zhao, Mingliang , Zhenxing Ren , Aihua Zhao , Yajun Tang , Junliang Kuang , Mengci Li , Tianlu Chen , et al. 2024. “Gut Bacteria‐Driven Homovanillic Acid Alleviates Depression by Modulating Synaptic Integrity.” Cell Metabolism 36: 1000–1012.e6. 10.1016/j.cmet.2024.03.010 38582087

[16] Sun, Yonggan , Qixing Nie , Shanshan Zhang , Huijun He , Sheng Zuo , Chunhua Chen , Jingrui Yang , et al. 2023. “Parabacteroides Distasonis Ameliorates Insulin Resistance via Activation of Intestinal GPR109a.” Nature Communications 14: 7740. 10.1038/s41467-023-43622-3 PMC10676405 38007572

[17] Zhou, Min , Lee J. Johnston , Chaodong Wu , and Xi Ma . 2023. “Gut Microbiota and Its Metabolites: Bridge of Dietary Nutrients and Obesity‐Related Diseases.” Critical Reviews in Food Science and Nutrition 63: 3236–3253. 10.1080/10408398.2021.1986466 34698581

[18] White, Phillip J. , Robert W. McGarrah , Mark A. Herman , James R. Bain , Svati H. Shah , and Christopher B. Newgard . 2021. “Insulin Action, Type 2 Diabetes, and Branched‐Chain Amino Acids: A Two‐Way Street.” Molecular Metabolism 52: 101261. 10.1016/j.molmet.2021.101261 PMC8513145 34044180

[19] Lynch, Christopher J. , and Sean H. Adams . 2014. “Branched‐Chain Amino Acids in Metabolic Signalling and Insulin Resistance.” Nature Reviews. Endocrinology 10: 723–736. 10.1038/nrendo.2014.171 PMC4424797 25287287

[20] Chen, Yimeng , Shujun Jiang , Haolu Wang , Mengzhen Si , Haixia Wu , Xinyi Liang , Shenmeng Yao , et al. 2025. “The Probiotic Parabacteroides Johnsonii Ameliorates Metabolic Disorders Through Promoting BCAAs to BSCFAs Conversion.” Advanced Science 12: e02624. 10.1002/advs.202502624 PMC12520484 40772426

[21] Ding, Ning , Hao Wu , Yiming Hua , Rui Hua , Bolin Li , Yifei Xie , Ying Xiong , et al. 2026. “Gut Microbiota‐Derived Isovaleric Acid Alleviates Atrial Fibrillation by Suppressing GSDME‐Dependent Pyroptosis.” Cell Metabolism 38: 370–387.e10. 10.1016/j.cmet.2025.12.017 41638192

[22] Choi, Jiyi , Moon Gyeong Yoon , Se Ha Jang , Geum Ok Baek , Hyun Sun Jung , Na‐Rae Lee , Choong Hwan Lee , et al. 2026. “Bacteroides Eggerthii Ameliorates Metabolic Dysfunction‐Associated Steatotic Liver Disease Through Host‐Microbe Signaling and Highlights 2‐Hydroxyisocaproate as a Potential Effector.” Clinical and Molecular Hepatology 32: 239–257. 10.3350/cmh.2025.0475 PMC12835788 41146521

[23] Holscher, Hannah D. 2017. “Dietary Fiber and Prebiotics and the Gastrointestinal Microbiota.” Gut Microbes 8: 172–184. 10.1080/19490976.2017.1290756 PMC5390821 28165863

[24] Gill, Samantha K. , Megan Rossi , Balazs Bajka , and Kevin Whelan . 2021. “Dietary Fibre in Gastrointestinal Health and Disease.” Nature Reviews. Gastroenterology & Hepatology 18: 101–116. 10.1038/s41575-020-00375-4 33208922

[25] Evans, Charlotte Elizabeth Louise . 2020. “Dietary Fibre and Cardiovascular Health: A Review of Current Evidence and Policy.” Proceedings of the Nutrition Society 79: 61–67. 10.1017/s0029665119000673 31266545

[26] Dodd, Dylan , Matthew H. Spitzer , William Van Treuren , Bryan D. Merrill , Andrew J. Hryckowian , Steven K. Higginbottom , Anthony Le , et al. 2017. “A Gut Bacterial Pathway Metabolizes Aromatic Amino Acids Into Nine Circulating Metabolites.” Nature 551: 648–652. 10.1038/nature24661 PMC5850949 29168502

[27] Deehan, Edward C. , Valentin Mocanu , and Karen L. Madsen . 2024. “Effects of Dietary Fibre on Metabolic Health and Obesity.” Nature Reviews. Gastroenterology & Hepatology 21: 301–318. 10.1038/s41575-023-00891-z 38326443

[28] Makki, Kassem , Edward C. Deehan , Jens Walter , and Fredrik Bäckhed . 2018. “The Impact of Dietary Fiber on Gut Microbiota in Host Health and Disease.” Cell Host & Microbe 23: 705–715. 10.1016/j.chom.2018.05.012 29902436

[29] Ross, Fiona C. , Dhrati Patangia , Ghjuvan Grimaud , Aonghus Lavelle , Eugene M. Dempsey , R Paul Ross , and Catherine Stanton . 2024. “The Interplay Between Diet and the Gut Microbiome: Implications for Health and Disease.” Nature Reviews Microbiology 22: 671–686. 10.1038/s41579-024-01068-4 39009882

[30] Wei, Wenchao , Chi Chun Wong , Zhongjun Jia , Weixin Liu , Changan Liu , Fenfen Ji , Yasi Pan , et al. 2023. “Parabacteroides Distasonis Uses Dietary Inulin to Suppress NASH via Its Metabolite Pentadecanoic Acid.” Nature Microbiology 8: 1534–1548. 10.1038/s41564-023-01418-7 PMC10390331 37386075

[31] Gomez‐Arango, Luisa F. , Helen L. Barrett , Shelley A. Wilkinson , Leonie K. Callaway , H David McIntyre , Mark Morrison , and Marloes Dekker Nitert . 2018. “Low Dietary Fiber Intake Increases Collinsella Abundance in the Gut Microbiota of Overweight and Obese Pregnant Women.” Gut Microbes 9: 189–201. 10.1080/19490976.2017.1406584 PMC6219589 29144833

[32] Liu, Ruixin , Jie Hong , Xiaoqiang Xu , Qiang Feng , Dongya Zhang , Yanyun Gu , Juan Shi , et al. 2017. “Gut Microbiome and Serum Metabolome Alterations in Obesity and After Weight‐Loss Intervention.” Nature Medicine 23: 859–868. 10.1038/nm.4358 28628112

[33] Wang, Zheng , Brandilyn A. Peters , Bing Yu , Megan L. Grove , Tao Wang , Xiaonan Xue , Bharat Thyagarajan , et al. 2024. “Gut Microbiota and Blood Metabolites Related to Fiber Intake and Type 2 Diabetes.” Circulation Research 134: 842–854. 10.1161/circresaha.123.323634 PMC10987058 38547246

[34] Yengo, Loic , Julia Sidorenko , Kathryn E. Kemper , Zhili Zheng , Andrew R. Wood , Michael N. Weedon , Timothy M. Frayling , et al. 2018. “Meta‐Analysis of Genome‐Wide Association Studies for Height and Body Mass Index in ∼700000 Individuals of European Ancestry.” Human Molecular Genetics 27: 3641–3649. 10.1093/hmg/ddy271 PMC6488973 30124842

[35] Fan, Yong , and Oluf Pedersen . 2021. “Gut Microbiota in Human Metabolic Health and Disease.” Nature Reviews. Microbiology 19: 55–71. 10.1038/s41579-020-0433-9 32887946

[36] Gomes, Aline Corado , Christian Hoffmann , and João Felipe Mota . 2018. “The Human Gut Microbiota: Metabolism and Perspective in Obesity.” Gut Microbes 9: 1–18. 10.1080/19490976.2018.1465157 PMC6219651 29667480

[37] Wei, Yahan , Dennise Palacios Araya , and Kelli L. Palmer . 2024. “ *Enterococcus faecium*: Evolution, Adaptation, Pathogenesis and Emerging Therapeutics.” Nature Reviews. Microbiology 22: 705–721. 10.1038/s41579-024-01058-6 PMC12147861 38890478

[38] Ng, Zhang Jin , Mazni Abu Zarin , Chee Keong Lee , Pongsathon Phapugrangkul , and Joo Shun Tan . 2020. “Isolation and Characterization of *Enterococcus faecium* DSM 20477 With Ability to Secrete Antimicrobial Substance for the Inhibition of Oral Pathogen *Streptococcus mutans* UKMCC 1019.” Archives of Oral Biology 110: 104617. 10.1016/j.archoralbio.2019.104617 31794906

[39] Mikeš, Z. , M. Ferenčík , E. Jahnová , L. Ebringer , I. Čižnár . 1995. “Hypocholesterolemic and Immunostimulatory Effects of Orally Applied Enterococcus faecium M‐74 in Man.” Folia Microbiologica 40: 639–646. 10.1007/BF02818522 8768254

[40] Liu, Zhi‐Lin , Yun‐Jiao Chen , Qing‐Lei Meng , Xin Zhang , and Xue‐Li Wang . 2023. “Progress in the Application of *Enterococcus faecium* in Animal Husbandry.” Frontiers in Cellular and Infection Microbiology 13: 1168189. 10.3389/fcimb.2023.1168189 PMC10437066 37600940

[41] Rangan, Kavita J. , Virginia A. Pedicord , Yen‐Chih Wang , Byungchul Kim , Yun Lu , Shai Shaham , Daniel Mucida , and Howard C. Hang . 2016. “A Secreted Bacterial Peptidoglycan Hydrolase Enhances Tolerance to Enteric Pathogens.” Science 353: 1434–1437. 10.1126/science.aaf3552 PMC5158264 27708039

[42] Yu, Haitao , Ganglian Lin , Junyan Jiang , Jiangqiao Yao , Zhenyan Pan , Haonan Xie , Zhiyuan Bo , et al. 2024. “Synergistic Activity of *Enterococcus faecium*‐Induced Ferroptosis via Expansion of IFN‐γ(+)CD8(+) T Cell Population in Advanced Hepatocellular Carcinoma Treated With Sorafenib.” Gut Microbes 16: 2410474. 10.1080/19490976.2024.2410474 PMC11445893 39353096

[43] Khalifa, Ashraf , and Hairul Islam Mohamed Ibrahim . 2023. “ *Enterococcus faecium* From Chicken Feces Improves Chicken Immune Response and Alleviates Salmonella Infections: A Pilot Study.” Journal of Animal Science 101: skad016. 10.1093/jas/skad016 PMC10011332 36651637

[44] Rashid, Muzamil , Anmol Narang , Shubham Thakur , Subheet Kumar Jain , and Sukhraj Kaur . 2023. “Therapeutic and Prophylactic Effects of Oral Administration of Probiotic *Enterococcus faecium* Smr18 in Salmonella enterica‐Infected Mice.” Gut Pathogens 15: 23. 10.1186/s13099-023-00548-x PMC10197331 37208771

[45] Liu, Xiaoning , Xiang Li , Bing Xia , Xin Jin , Qianhui Zou , Zhenhua Zeng , Weiyang Zhao , et al. 2021. “High‐Fiber Diet Mitigates Maternal Obesity‐Induced Cognitive and Social Dysfunction in the Offspring via Gut‐Brain Axis.” Cell Metabolism 33: 923–938.e6. 10.1016/j.cmet.2021.02.002 33651981

[46] Deng, Jie , Kai Zhou , Caimin Feng , Yilu Bao , Zhiming Zhang , Wenfeng Luo , and Meiying Li . 2024. “Effect of Konjac Glucomannan on Gut Microbiota From Hyperuricemia Subjects In Vitro: Fermentation Characteristics and Inhibitory Xanthine Oxidase Activity.” Frontiers in nutrition 11: 1465940. 10.3389/fnut.2024.1465940 PMC11446875 39364150

[47] Zhu, Sijia , Jiyu Yang , Pengkui Xia , Sha Li , Qi Wang , Kaikai Li , Bin Li , and Jing Li . 2024. “Effects of Konjac Glucomannan Intake Patterns on Glucose and Lipid Metabolism of Obese Mice Induced by a High Fat Diet.” Food & Function 15: 9116–9135. 10.1039/D4FO02442G 39219450

[48] Mao, YuHeng , YiXuan Xu , YanHeng Li , Jing Cao , FengLing Song , Dan Zhao , Yimin Zhao , Zhao‐Mei Wang , and Yan Yang . 2021. “Effects of Konjac Glucomannan with Different Molecular Weights on Gut Microflora With Antibiotic Perturbance in In Vitro Fecal Fermentation.” Carbohydrate Polymers 273: 118546. 10.1016/j.carbpol.2021.118546 34560958

[49] Mao, YuHeng , Yixuan Xu , Fenglin Song , ZhaoMei Wang , YanHeng Li , Mingzhu Zhao , Fang He , Zezhong Tian , and Yan Yang . 2022. “Protective Effects of Konjac Glucomannan on Gut Microbiome With Antibiotic Perturbation in Mice.” Carbohydrate Polymers 290: 119476. 10.1016/j.carbpol.2022.119476 35550768

[50] Fan, Lijuan , Yaoyao Xia , Youxia Wang , Dandan Han , Yanli Liu , Jiahuan Li , Jie Fu , et al. 2023. “Gut Microbiota Bridges Dietary Nutrients and Host Immunity.” Science China Life Sciences 66: 2466–2514. 10.1007/s11427-023-2346-1 PMC10247344 37286860

[51] Mao, Bingyong , Dongyao Li , Jianxin Zhao , Xiaoming Liu , Zhennan Gu , Yong Q. Chen , Hao Zhang , and Wei Chen . 2014. “In Vitro Fermentation of Lactulose by Human Gut Bacteria.” Journal of Agricultural and Food Chemistry 62: 10970–10977. 10.1021/jf503484d 25340538

[52] Mao, Bingyong , Hongyu Tang , Jiayu Gu , Dongyao Li , Shumao Cui , Jianxin Zhao , Hao Zhang , and Wei Chen . 2018. “In Vitro Fermentation of Raffinose by the Human Gut Bacteria.” Food & Function 9: 5824–5831. 10.1039/c8fo01687a 30357216

[53] Takeuchi, Tadashi , Keishi Kameyama , Eiji Miyauchi , Yumiko Nakanishi , Takashi Kanaya , Takayoshi Fujii , Tamotsu Kato , et al. 2023. “Fatty Acid Overproduction by Gut Commensal Microbiota Exacerbates Obesity.” Cell metabolism 35: 361–375.e9. 10.1016/j.cmet.2022.12.013 36652945

[54] Sakko, M. , L. Tjäderhane , T. Sorsa , P. Hietala , A. Järvinen , P. Bowyer , and R. Rautemaa . 2012. “2‐Hydroxyisocaproic Acid (HICA): A New Potential Topical Antibacterial Agent.” International Journal of Antimicrobial Agents 39: 539–540. 10.1016/j.ijantimicag.2012.02.006 22483561

[55] Sakko, Marjut , Riina Rautemaa‐Richardson , Samuli Sakko , Malcolm Richardson , and Timo Sorsa . 2021. “Antibacterial Activity of 2‐Hydroxyisocaproic Acid (HICA) Against Obligate Anaerobic Bacterial Species Associated With Periodontal Disease.” Microbiology Insights 14: 11786361211050086. 10.1177/11786361211050086 PMC8543563 34707364

[56] Zhang, Yi , Xuemei Wang , Jun Lin , Jia Liu , Kai Wang , Qixing Nie , Chuan Ye , et al. 2024. “A Microbial Metabolite Inhibits the HIF‐2α‐Ceramide Pathway to Mediate the Beneficial Effects of Time‐Restricted Feeding on MASH.” Cell Metabolism 36: 1823–1838.e6. 10.1016/j.cmet.2024.07.004 39079531

[57] Georgiev, Asen , Cesare Granata , and Michael Roden . 2022. “The Role of Mitochondria in the Pathophysiology and Treatment of Common Metabolic Diseases in Humans.” American Journal of Physiology‐Cell Physiology 322: C1248–C1259. 10.1152/ajpcell.00035.2022 35508191

[58] Gopal, Raj K. , Venkata R. Vantaku , Apekshya Panda , Bryn Reimer , Sneha Rath , Tsz‐Leung To , Adam S. Fisch , et al. 2023. “Effectors Enabling Adaptation to Mitochondrial Complex I Loss in Hürthle Cell Carcinoma.” Cancer Discovery 13: 1904–1921. 10.1158/2159-8290.Cd-22-0976 PMC10401073 37262067

