# Supplementary material for: Gut Enterococcus faecium derived leucic acid mediates the antiobesity effect of dietary fiber
Source: Imeta. 2026 Sep 21;5(5):e70173. doi: 10.1002/imt2.70173 (PMC13592376; doi:10.1002/imt2.70173)
Supplement: Supplementary file 1 [file IMT2-5-e70173-s002.docx]

**Supporting information to Gut *Enterococcus faecium* derived leucic acid mediates the antiobesity effect of dietary fiber**

**Running title: *E. faecium* derived LEA counteracts obesity**

Baifei Hu^1,2,#^, Qianru Gao^1,#^, Junmin Hui^1^, Mengqiong Wang^1^, Haiyan Gao^1,2^, Kai Li^1,2^, Junping Zheng^1,2^, Zhe Dai^3,*^, Songlin Liu^1,2,*^, Hongtao Liu^1,2,*^

^1^College of Basic Medical Sciences; Key Laboratory of Chinese Medicinal Resource and Chinese Herbal Compound of the Ministry of Education, Hubei University of Chinese Medicine, Wuhan 430065, PR China.

^2^Hubei Shizhen Laboratory, Wuchang District Huayuanshan 4, Wuhan 430061, PR China.

^3^Zhongnan Hospital of Wuhan University, East Lake Road, Wuchang District, Wuhan, 430071, PR China

^#^These authors contributed equally: Baifei Hu, Qianru Gao

^*^Correspondence: daizhe@znhospital.cn (Zhe Dai), [newforest@163.com](mailto:newforest@163.com) (Songlin Liu), [hongtaoliu@hbucm.edu.cn](mailto:hongtaoliu@hbucm.edu.cn) (Hongtao Liu).

**Supplementary Figures**

**Figure S1.** Analysis of gut microbiota diversity and differential species between healthy and obese groups.

**Figure S2.** Genomic characteristics and functional annotation of the two isolated *E.* *faecium* strains.

**Figure S3.** Validation of strain-specific detection and metabolic effects following *E. faecium* colonization.

**Figure S4.** *E.* *faecium* intervention restored hepatic mitochondrial energy metabolism in HFD-fed mice.

**Figure S5.** LEA was a candidate bioactive metabolite produced by *E.* *faecium*.

**Figure S6.** LEA alleviated hepatic pathological damage and improved mitochondrial function in HFD-fed mice.

**Figure S7**. High-content screening of AML12 cells and TLC analysis of fermentation supernatants.

**Figure S8.** GM improved HFD-induced obesity in mice and enriched *E. faecium* *in vivo*.

**Figure S9.** GM ameliorated metabolic disorders in obese mice.

**Figure S10.** Alleviation of obesity by GM was microbiota-dependent.

**Supplementary Methods**

**Method S1:** Whole-genome sequencing of two *E. faecium* strains isolated from human and mouse feces, respectively.

**Method S2:** Method for detection of human fecal and dietary fiber co-incubated samples *in vitro*.

**Method S3:** Establishment of an antibiotic mouse model.

**Method S4:** Procedure of Oil Red O staining.

**Method S5:** Preparation of culture medium supernatant with different molecular weight.

**Method S6:** Non-target metabolomics analysis for bacteria culture supernatant.

**Method S7:** AML12 cell culture and treatment.

**Method S8:** Glucose tolerance test (GTT).

**Method S9:** 16S rDNA gene sequencing and analysis.

**Method S10:** Detection of mitochondrial membrane potential and morphology.

**Method S11:** Western blotting.

**Method S12:** High-content screening.

**Method S13:** Metagenomic sequencing.

**Method S14:** Proteomic analysis.

**Supplementary Figures**

**Figure S1. Analysis of gut microbiota diversity and differential species between healthy and obese groups.** (A) α-diversity of gut microbiota in healthy and obese groups (Chao1 and ACE indices). (B) Non-metric multidimensional scaling (NMDS) analysis of gut microbiota β-diversity. (C) Orthogonal partial least squares discriminant analysis (OPLS-DA) of gut microbiota between the two groups. (D) Heatmap of relative abundance of differential bacterial species at the species level. (E) Volcano plot of differentially abundant species between the two groups. (F) Variable importance in projection (VIP) scores. Data were expressed as mean ± SD. ^**^*p* < 0.01 *vs.* healthy group.

**Figure S2. Genomic characteristics and functional annotation of the two isolated *Enterococcus faecium* strains.** (A, C) Circular genome maps of *Ef*-H (A) and *Ef*-M (C), displaying GC content, coding sequences (CDSs), non-coding RNA regions (ncRNA, rRNA, tRNA), and GC skew. (B, D) Top 20 KEGG pathway enrichment analysis of genes from *Ef*-H (B) and *Ef*-M (D), with the x-axis representing the number of genes assigned to each pathway. (E) CAZy classification of *Ef-*M. (F) CAZy classification of *Ef*-H.

**Figure S3. Validation of strain-specific detection and metabolic effects following *E. faecium* colonization.** (A, B) Validation of strain-specific primers for mouse-derived *E. faecium* (A) and human-derived *E. faecium* (B) via serial dilution assays. (C) Colonization levels of target strains in fecal samples of mice in each group. (D) Body weight change curve. (E) Body weight gain. (F) Western blot images of p-ACC, ACC, SREBP1, p-AMPK, AMPK, PPARα. (G) Quantification of relative protein expression. Data were expressed as mean ± SD. *^*^p* < 0.05, *^**^p* < 0.01 *vs.* NCD group, ^#^*p* < 0.05, ^##^*p* < 0.01 *vs.* HFD group. L*Ef*-M, live mouse-derived *E. faecium*; L*Ef*-H, live human-derived *E. faecium*.

**Figure S4. *E. faecium* intervention restored hepatic mitochondrial energy metabolism in HFD-fed mice.** (A) Activity of hepatic mitochondrial respiratory chain complex I. (B) Hepatic ATP content in the four groups. Data were expressed as mean ± SD. ^*^*p* < 0.05, ^**^*p* < 0.01 *vs.* NCD group; ^#^*p* < 0.05, ^##^*p* < 0.01 *vs.* HFD group.

**Figure S5.** **LEA was a candidate bioactive metabolite produced by *E. faecium*.** (A) Orthogonal partial least squares discriminant analysis (OPLS-DA) score plot of untargeted metabolomics. (B) Heatmap of differential metabolites. (C) Principal Component Analysis (PCA) score plot of proteomics. (D) OPLS-DA score plot of proteomics. (E) Heatmap of branched-chain amino acids (BCAAs)-related gene abundance in gut microbiota from healthy *vs.* obese individuals.

**Figure S6. LEA alleviated hepatic pathological damage and improved mitochondrial function in HFD-fed mice.** (A) Body weight curve. (B) Body weight gain. (C) Indices of epididymal, mesenteric, subcutaneous, and brown adipose tissues. (D) Gross morphology of the liver (left), hematoxylin and eosin (H&E) staining (middle), and Oil Red O staining (right) in mice. Scale bar = 50 µm or 100 µm. (E) Activity of hepatic mitochondrial complex I. (F) Hepatic ATP content. (G) Western blot images of p-ACC, ACC, SREBP1, p-AMPK, AMPK, PPARα. (H) Quantification of relative protein expression. Data were expressed as mean ± SD. ^*^*p* < 0.05, ^**^*p* < 0.01 *vs.* NCD group; ^#^*p* < 0.05, ^##^*p* < 0.01 *vs.* HFD group.

**Figure S7.** **High-content screening of AML12 cells and TLC analysis of fermentation supernatants.** (A) High-content fluorescence imaging and quantitative analysis of intracellular lipid droplets in PA-induced AML12 hepatocytes after treatment with fermentation supernatants of human-derived *E. faecium* combined with different dietary fibers. Nuclei were stained with blue fluorescence, and lipid droplets were stained with green fluorescence. Statistical histogram showing the quantitative proportion of mean lipid droplet area per cell in each group. Scale bar = 50 µm. (B) Representative thin layer chromatography (TLC) of fermentation supernatants after 24 h incubation of dietary fiber with human feces. GG, guar gum; GM, Glucomannan.

**Figure S8.** **GM improved HFD-induced obesity in mice and enriched *E. faecium in vivo*.** (A) Schematic diagram. (B) Glucose tolerance test curves. (C) Area under the curve of glucose tolerance test. (D) Weight of subcutaneous adipose, mesenteric adipose, and epididymal adipose. (E) Concentrations of cholesterol, triglyceride, and LDL-C*.* (F) H&E staining and Oil Red O staining images of liver and mesenteric adipose tissues*.* Scale bar = 50, 100, and 200 µm (G) Shannon index. (H) Simpson index. (I) Partial least squares Discriminant Analysis (PLS-DA). (J) The relative abundance of fecal *Enterococcus*. (K) Linear discriminant analysis effect size (LEfSe). (L) Linear Discriminant Analysis (LDA). Data were expressed as mean ± SD (n = 6). ^*^*p* < 0.05, ^**^*p* < 0.01 *vs.* NCD group; ^#^*p* < 0.05, ^##^*p* < 0.01 *vs.* HFD group. LDL-C, low-density lipoprotein cholesterol.

**Figure S9.** **GM ameliorated metabolic disorders in obese mice.** (A) Body weight curve. (B) Body weight gain. (C) Concentration of fasting blood glucose. (D) Relative mRNA expression of factors associated with lipid metabolism. (E) Non-metric Multidimensional Scaling (NMDS). Data were expressed as mean ± SD (n = 6). ^*^*p* < 0.05, ^**^*p* < 0.01 *vs.* NCD group; ^#^*p* < 0.05, ^##^*p* < 0.01 *vs.* HFD group. NCD, normal chow diet; HFD, high-fat diet; *Pparα*, proliferator-activated receptor α; *Cebp1*, CCAAT enhancer-binding protein 1; *Fasn*, fatty acid synthase; *Srebp1*, sterol regulatory element-binding protein 1.

**Figure S10. Alleviation of obesity by GM was microbiota-dependent.** (A) Schematic diagram. (B) Body weight. (C) Concentration of fasting blood glucose. (D−F) Concentrations of cholesterol, triglyceride, and LDL-C. (G) Chao1 index. (H) Observed species index. (I) Non-metric multidimensional scaling analysis (NMDS). (J) Principal component analysis (PCA). (K) Relative abundance at genus level. (L) Heatmap of characterized gut microbiota at the genus level. Data were expressed as means ± SD (n = 6). ^*^*p* < 0.05, ^**^*p* < 0.01 *vs.* HFD group. HFD, high-fat diet; ABx, Antibiotic mixtures; LDL-C, low-density lipoprotein cholesterol.

**METHODS**

1. **Whole-genome sequencing of two *E. faecium* strains isolated from human and mouse feces, respectively**

Genomic DNA was extracted using the Monarch Spin gDNA Extraction Kit (T3010, New England Biolabs), and quantified using an Invitrogen Qubit 4 Fluorometer. Sequencing libraries were prepared using the Rapid Barcoding Kit 24 V14 (SQK-RBK114.24, ONT) and sequenced on a PromethION 2 Integrated platform with R10.4.1 flow cells (FLO-PRO114M, ONT) under the control of MinKNOW software. Besides, the specific primer sequences for *E. faecium* were referenced from the previous research [1].

2. **Method for detection of human fecal and dietary fiber co-incubated samples *in vitro***

To detect the degradation ability of human gut microbiota to five kinds of dietary fibers, we determined the total sugar content of the collected samples by the phenol-sulfate method and TLC. A 5 μL medium was carefully loaded onto a pre-coated silica gel-60 aluminum TLC plate (1055540001, Merck, Darmstadt). The samples were resolved using the freshly prepared n-butanol/formic acid/water (4∶6∶1, v/v/v) solution as the eluent. After that, the loaded plate was immersed into the 10% H_2_SO_4_ (v/v) in ethanol (containing phenol), then the spots on the TLC were detected after heating at 120 ℃ for 5 min.

3. **Establishment of an antibiotic mouse model**

To establish an antibiotic mouse model, an antibiotic mixture (ABx) dissolved in drinking water (1 mg/mL neomycin sulfate (N109017, Aladdin, China), 1 mg/mL ampicillin (A105484, Aladdin, China), 0.5 mg/mL vancomycin (V105495, Aladdin, China), and 0.5 mg/mL metronidazole (M109874, Aladdin, China) was used for 3 months to remove the gut microbiota. During the experiment, male C57BL/6J mice were given HFD and 1 mg/mL GM dissolved in drinking water.

4. **Procedure of Oil Red O staining**

The sections were fixed with paraformaldehyde for 10 min, then washed twice with PBS and immersed in 60% isopropyl alcohol for 20 s. Next, we added freshly made Oil Red O (G1260, Solarbio, China), dyed for 30 min, and removed the dye and non-specific staining with 60% isopropyl alcohol. The nucleus was stained with hematoxylin (DC0045, Solarbio, China), and the slides were sealed with glycerin gelatin.

5. **Preparation of culture medium supernatant with different molecular weight**

Mouse-derived *Enterococcus faecium* in the logarithmic growth stage was inoculated into BHI medium at a ratio of 5%. After anaerobic culture at 37 °C for 24 hours, the medium was collected and centrifuged at 4000 rpm for 15 min. The supernatant was filtered and sterilized by 0.22 μm membrane. The supernatant was then placed in an Amicon Ultra-15 Centrifugal Filter Units (UFC9003, Millipore, Darmstadt) with a pore size of 3 kD and centrifuged at 4000 ×g for 45 min. After that, the solution was divided into a > 3 kD fraction and a < 3 kD fraction.

6. **Non-target metabolomics analysis for bacteria culture supernatant**

Following a 24 h fermentation of mouse-derived *Enterococcus faecium* in basal and nutrient (contained GM) media, 200 μL of fermentation broth was individually collected. And added 800 μL methanol separately to fermentation broths, then sonicated in an ice bath for 20 min. The supernatant was removed and passed through a 0.22 μm filter membrane for LC-MS analysis. The LC-MS was carried out using Agilent G6545 LC-QTOF, Dual AJS ESI ion source, and automatic sampler. The chromatographic separation was carried out on a ZORBAX SB-C18 column (2.1 × 100 mm 1.8-Micron) at a column temperature of 35 °C. The injection volume was 2 μL, and the mobile phase was composed of 0.1% formic acid in water (A) and methanol (B) at a flow rate of 0.3 mL/min. The analysis was carried out with an elution gradient as follows: 0‒1 min, 5% B; 1‒24 min, 5% B‒100% B; 24‒28 min, 100% B; 28‒28.5 min, 100% B‒5% B; 28.5‒30 min, 5% B. The mass spectrometry analysis was performed in both positive and negative ion modes. Ion source parameters were as follows: dry gas temperature: 320 °C; drying gas (N_2_) flow rate, 8 L/min; nebuliser pressure, 35 psi; sheath gas temperature, 350 °C; sheath gas flow rate, 11 L/min; capillary voltage: +3500 V/-3500 V. Auto MS/MS mode scanning mass ranges: parent ion mass scanning ranged from 100‒1500 m/z, The scanning mass range for fragment ions was 50‒1000 m/z. Data was analyzed using Agilent Masshunter Qualitative Analysis software combined with the databases Metlin-AM-PCDL, Metlin-AMRT-PCDL, Metlin-Lipids-AM-PCDL, Metlin-Metabolites-AM-PCDL, Metlin-Peptides-AM-PCDL for compositional identification.

7. **AML12 cell culture and treatment**

AML12 cell (CRL-2254, ATCC) was cultured in DME/F12 medium (SH30023.01, HyClone, Utah) supplemented with 10% fetal bovine serum (FBS) (11011-8611, Tianhang, China), 1% insulin–transferrin–selenium (C0341, Beyotime, China), 40 ng/mL dexamethasone (ST1258, Beyotime, China), and 1% penicillin-streptomycin (C0222, Beyotime, China) at 37  °C under 5% CO_2_. To simulate *in vivo* hepatic steatosis, AML12 cells were maintained in a medium containing 200 μM palmitic acid (PA) (P5585, Sigma-Aldrich, Darmstadt) for 12 h. To evaluate the effects of high molecular weight (HMW), low molecular weight (LMW), and LEA on PA-induced lipid metabolism in AML12 cells, the cells were co-treated with PA and each of the test compounds (LEA, HMW, or LMW) for 12 h. We then collected the cells for lipid droplet green fluorescence detection (C2053, Beyotime, China), qPCR analysis, and TG content determination.

8. **Glucose tolerance test (GTT)**

GTT was assayed at the end of the animal experiment. The mice were fasted for 12 hours overnight and then injected with 1 g/kg glucose intraperitoneally. Blood glucose was measured by glucose meter at 0, 15, 30, 60, 120, and 180 min after glucose injection.

9. **16S rDNA gene sequencing and analysis**

DNA for gut microbiota analysis was extracted from 100 mg of cecum contents of obese mice treated with GM. The V3−V4 region of 16S rDNA gene was amplified. Purified amplicons were subjected to paired-end sequencing (2 × 300) on the Illumina MiSeq platform (Illumina, San Diego, CA, USA) at Allwegene Tech (Beijing, China). Sequences were clustered into operational taxonomic units (OTUs) with greater than 97% similarity using UCLUST (version 1.2.22). UCLUST was performed using Silva (Release 119) and the Greengene 16S rDNA database (Release 13.5, http://www.greengenes. secondgenome.com) for each 16S rRNA gene sequence classification with a minimum confidence level of 90%. Finally, OTU tables were generated for gut microbiota composition and abundance analysis.

10. **Detection of mitochondrial membrane potential and morphology**

AML12 cells were seeded on cell culture slides and co-treated with PA and various concentrations of LEA for 24 h. After the cells were processed, the cells were collected and resuspended for counting. 1× 10^6^ cells were taken, centrifuged at 300 ×g for 5 min, and after discarding the supernatant, the cells were resuspended by adding 500 μL of JC-1 working solution (C2003, Beyotime, China), and incubated at 37 °C for 20 min under light protection. The cells were rinsed with JC-1 Assay Buffer, and the cells were resuspended by centrifugation and discarding the supernatant, and then the cells were immediately detected on the flow cytometry. The mitochondrial membrane potential level was expressed as the ratio of the Q2 to the Q3, and the experiment was repeated three times.

Additionally, AML12 cells were seeded in confocal dishes and co-treated with PA and various concentrations of LEA for 24 h. Following PBS washing, the cells were incubated with 50 nM Mito-Tracker Red CMXRos (C1035, Beyotime, China) and Hoechst 33342 (C1025, Beyotime, China) for 20 min, respectively. Images were then acquired using a Leica confocal microscope.

11. **Western blotting**

The antibodies used were as follows: p-ACC (3661, CST, Massachusetts), ACC (3662, CST, Massachusetts), p-AMPK (2535, CST, Massachusetts), AMPK (2603, CST, Massachusetts), SREBP1 (PAB39550, Bioswamp, China), and β-Actin (ZB15001-HRP-100, Servicebio, China). The densitometry analysis was performed using Image J2x software (v1.8.0).

12. **High-content screening**

We prepared solutions of dietary fibers including pectin (P112756, Aladdin, China), inulin (S11143, Yuanye, China), GM (P-GICML, MEGAZYME, Wicklow), fructooligosaccharides (FOS) (F303397, Aladdin, China), guar gum (GG) (G4129, Merck, Darmstadt), galactooligosaccharides (GOS) (S11138, Yuanye, China), and fucoidan (FUC) (F304947, Aladdin, China) at a concentration of 2.5 mg/mL. After sterilization at 121 °C for 20 minutes, *E. faecium* was inoculated into each of the above dietary fiber solutions at a 5% ratio for fermentation. The fermentation broth was collected, centrifuged at 12000 × g to obtain the supernatant, and filtered to remove bacteria. AML12 cells were cultured in a medium containing 200 μM palmitic acid (PA) (P0500, Merck, Darmstadt) and 5% of the above fermentation supernatant for 12 hours. Subsequently, the lipid droplet green fluorescence assay kit was used to fluorescently stain lipid droplets in AML12 cells, followed by evaluation of lipid droplet area using a high-content screening system.

13. **Metagenomic sequencing**

Total DNA was extracted from fecal samples using the E.Z.N.A.® Stool DNA Kit (Omega Bio-tek, Norcross, GA, U.S.) according to manufacturer’s protocols. Metagenomic shotgun sequencing libraries were constructed and sequenced on an Illumina or MGI-T7 platform with PE150 strategy. Raw reads were quality-controlled using Trimmomatic to remove adapters and low-quality sequences. Host-derived reads were filtered by mapping to the human reference genome (NCBI) with BWA-MEM. The resulting clean reads from each sample were assembled de novo using MEGAHiT (--min-contig-len 500). Open reading frames were predicted from contigs using Prodigal (v2.6.3) and clustered into a non-redundant gene set with CD-HIT (identity ≥95%, coverage ≥90%). Gene abundances were quantified using Salmon and normalized as reads per kilobase per million. For functional annotation, protein sequences of non-redundant genes were aligned against multiple databases, including eggNOG, NR, KEGG (using KofamScan), CAZy (using HMMER), and specialized datasets such as SARG, VFDB, and PHI, with DIAMOND (e-value < 0.00001, identity ≥ 70%). Alpha diversity (Shannon index, richness) and beta diversity (Bray-Curtis distance) were calculated using MOTHUR and the R vegan package, respectively.

14. **Proteomic analysis**

Mouse liver samples or *E. faecium* were thoroughly lysed using an SDS/Tris-HCl lysis buffer. Following protein denaturation, reduction, alkylation, digestion, peptide cleanup, and vacuum drying, the resulting peptide powders were reconstituted in 0.1% formic acid. The samples were then analyzed using an ultra-high-resolution mass spectrometer (Orbitrap Astral) operating in data-dependent acquisition (DDA) mode, which automatically switched between MS and MS/MS scans. A PepMap RSLC C18 column (2 μm, 100 Å, 150 μm × 15 cm, Thermo Fisher Scientific) was used for peptide separation. The raw DDA data were processed and analyzed using Spectronaut 16.0 software (Biognosys AG, Switzerland) with default settings to generate a candidate library. Protein quantification was calculated based on the sum of the top 3 filtered peptides at a 1% FDR threshold.

**Reference**

1. Maheux, Andrée F, Luc Bissonnette, Maurice Boissinot, Jean-Luc T Bernier, Vicky Huppé, François J Picard, Éve Bérubé, Michel G Bergeron. 2011. “Rapid concentration and molecular enrichment approach for sensitive detection of Escherichia coli and Shigella species in potable water samples.” *Applied and environmental microbiology* 77: 6199−6207. <https://doi.org/10.1128/aem.02337-10>
